# Enrichment Map: A Network-Based Method for Gene-Set Enrichment Visualization and Interpretation

**DOI:** 10.1371/journal.pone.0013984

**Published:** 2010-11-15

**Authors:** Daniele Merico, Ruth Isserlin, Oliver Stueker, Andrew Emili, Gary D. Bader

**Affiliations:** Department of Molecular Genetics, Banting and Best Department of Medical Research, Donnelly Centre for Cellular and Biomolecular Research, University of Toronto, Toronto, Ontario, Canada; King Abdullah University of Science and Technology, Saudi Arabia

## Abstract

**Background:**

Gene-set enrichment analysis is a useful technique to help functionally characterize large gene lists, such as the results of gene expression experiments. This technique finds functionally coherent gene-sets, such as pathways, that are statistically over-represented in a given gene list. Ideally, the number of resulting sets is smaller than the number of genes in the list, thus simplifying interpretation. However, the increasing number and redundancy of gene-sets used by many current enrichment analysis software works against this ideal.

**Principal Findings:**

To overcome gene-set redundancy and help in the interpretation of large gene lists, we developed “Enrichment Map”, a network-based visualization method for gene-set enrichment results. Gene-sets are organized in a network, where each set is a node and edges represent gene overlap between sets. Automated network layout groups related gene-sets into network clusters, enabling the user to quickly identify the major enriched functional themes and more easily interpret the enrichment results.

**Conclusions:**

Enrichment Map is a significant advance in the interpretation of enrichment analysis. Any research project that generates a list of genes can take advantage of this visualization framework. Enrichment Map is implemented as a freely available and user friendly plug-in for the Cytoscape network visualization software (http://baderlab.org/Software/EnrichmentMap/).

## Introduction

High-throughput genomic experiments often lead to the identification of large gene lists [1], [2], [3]. Gene lists are typically defined using statistical methods appropriate to the experimental design. For instance, a frequently applied method is to score genes by their differential expression between two biological states (such as healthy vs. diseased). Especially in more mature fields, like gene expression microarrays, the statistical models used for gene scoring are well established [1], [4]. For instance, gene expression values are ranked to identify the top-most list of expressed genes, based on an arbitrary expression threshold, or a set of gene expression experiments are clustered, each cluster defining a potentially large gene list. However, these methods for finding interesting genes often do not help the interpretation of the resulting gene lists and the formulation of consistent biological hypotheses from these results still poses a major challenge for experimentalists. Searching for sets of predefined functionally related genes (e.g. pathways) that are enriched in a gene list is a popular method designed to solve this problem. However, as gene-set collections get larger and more complex, users may experience longer lists of results and increased redundancy between sets. We have developed a visualization method for gene set enrichment results, called Enrichment Map, which helps quickly find general functional themes in genomics data. In the next sections, we introduce enrichment analysis, the gene set redundancy problem and then explain how Enrichment Map works using typical analysis scenarios.

### Enrichment analysis

Early approaches to gene list interpretation relied on choosing a handful of high scoring genes, and then building rather subjective, anecdotal interpretations. Enrichment analysis is an automated and statistically rigorous technique to analyze and interpret large gene lists using *a priori*-knowledge [5]. Enrichment analysis assesses the over- (or under-) representation of a known set of genes (e.g. a biological pathway) within the input gene list [6], [7], [8]. If a statistically significant number of genes from the known set are present in the gene list, it may indicate that the biological pathway plays a role in the biological condition under study. This analysis is repeated for all available known gene-sets, which could number in the thousands.

Over 60 enrichment analysis methods and tools have been developed in the last few years [5], [9], [10]. They mainly differ in (a) their database of known gene-sets and (b) the statistical method used to assess enrichment. In the following paragraphs, we briefly review existing approaches for enrichment analysis considering these two facets.

Most enrichment tools derive gene-sets from Gene Ontology (GO) annotations [11], because they are readily accessible for many organisms and cover many genes, yet many other sources of gene-sets exist and are used by some tools in addition to GO [7], [12], [13]. Gene-sets can be defined based on participation in a metabolic or signaling pathway (e.g. KEGG [14], Reactome [15]), targeting by gene expression regulators (e.g. microRNA, transcription factors), protein features such as domains, chromosomal location and association to specific diseases, stimuli, or genetic perturbations. Gene-sets from multiple sources are collected in resources such as MSigDB [13] or WhichGenes [16]. Not all organisms are well covered by gene-sets and many tools only support specific organisms.

Statistical methods to determine enrichment are usually either threshold-dependent or whole-distribution [9]. Threshold-dependent techniques require the user to input a discrete list of top-ranking genes, which may require setting a threshold on the gene scoring statistic. The one-tail *Fisher's Exact Test* [17], based on the *hypergeometric distribution*, was the first method proposed to address this problem [18], and continues to be one of the most used testing methods of this type [19]. These methods are useful for naturally discrete lists, but have major drawbacks when utilized with continuous gene scores. Specifically, results may not be stable to choice of threshold [20], and there is loss of information caused by treating gene scores in a binary way (they either pass the threshold or not). On the other hand, whole-distribution methods are threshold-free, as they test gene-sets by comparing their score distribution versus the background distribution. For this reason they are often preferred over threshold-dependent methods for gene lists associated with a continuous score. GSEA (Gene-Set Enrichment Analysis) [13], which utilizes the gene rank derived from differential expression or other statistics, is one of the most popular techniques in this group, though other whole-distribution testing models have been proposed [5], [9].

### The gene-set redundancy problem

The growing number of available gene-sets, due to the increased availability of functional annotations, makes enrichment analysis a powerful tool to help researchers gain interesting insights from their high-throughput data. However, this comes at a cost: as gene-set collections get larger and more complex, there may be longer lists of results and increased redundancy between sets. Redundancy is particularly problematic with gene-sets derived from hierarchical functional annotation systems, like GO, as children terms are partially redundant with their parents by definition. Gene-set redundancy constitutes a major barrier for the interpretability of enrichment results, limiting the full exploitation of its analytic power.

This problem can be addressed by modifying either the statistical test or the gene-sets to minimize the effect of redundancy and produce more concise enrichment results. Existing methods usually take advantage of the hierarchical structure of Gene Ontology to reduce redundancy, a solution that is only effective for GO or other hierarchically organized gene sets and not applicable to many others, such as pathways, experimental signatures and regulator targets. POSOC [21] exploits the GO hierarchy to merge single sets into clusters, which can then be tested for enrichment. Ontologizer [22] defines a modified *Fisher's Exact Test* for hierarchical vocabularies, termed the *parent-child approach*. Enrichment of a given gene-set is calculated with respect to the parent gene-set, instead of the list of genes in the experiment (the experimental universe set), in order to downplay uninformative child enrichment (i.e. enrichment merely “inherited” from the parent set enrichment). GOstats [23] and elim [24] adopt a reverse strategy: child terms (i.e. leaves in the hierarchy) are tested first, then parent nodes are modified so as not to include the genes present in their enriched children. Ontologizer tends to penalize smaller gene-sets, whereas GOstats and elim tend to penalize larger gene-sets. This problem is overcome by the weight algorithm [24], which reweights genes based on how many children gene sets they are part of, but this is however limited to hierarchical vocabularies and requires using *Fisher's Exact Test*.

If test and gene-set modification methods are not completely satisfactory solutions to the gene-set redundancy problems, what else is available? A different strategy relies on visualizing the redundancy relations among gene-sets to help the user recognize redundancy while they explore enrichment results. Tools such as Onto-Express [25], the Cytoscape [26] plugin BiNGO [27] and WebGestalt [28] display the hierarchical structure of enriched GO terms. This helps identify parent-child relationships between terms, but the applicability is again confined to hierarchical vocabularies. Other tools are more flexible. They neglect any a-priori gene-set structure and compute a similarity score among gene-sets, capturing inter gene-set redundancy. DAVID [12], [29], [30] utilizes fuzzy gene clusters, pre-computed on the basis of annotation similarity among all genes, to sort enriched gene-sets into different yet partially overlapping groups; results are then displayed in a tabular format. Molecular Concept Maps (MCM) software [31] and the ClueGO [32] Cytoscape plugin offer a richer visualization solution than DAVID, displaying enriched gene-sets as a network, where each gene-set is represented as a node and edges connect similar gene-sets. MCM utilizes the *Fisher's Exact Test* p-value as a similarity score between gene-sets. The MCM network includes the input gene list together with the enriched gene-sets. Colors are used to differentiate the gene-set sources. ClueGO determines gene-set similarity according to Cohen's kappa statistic. Gene-sets are then clustered using an iterative merging approach; nodes, representing enriched gene-sets, are colored according to cluster membership or alternatively according to the proportion of up- and down-regulated genes; node size represents enrichment significance. ClueGO and MCM are useful, as they offer an expressive and intuitive organization of gene-sets, applicable to any gene-set source. Unfortunately, all the tools mentioned so far incorporate only one enrichment test (the *Fisher's Exact Test*) and are not designed to work with enrichments computed using other methods, limiting their power and flexibility. Many tools only use GO annotation as a source of gene-sets, and do not take advantage of the many types of other useful gene-sets that exist. The landscape of available solutions to gene-set redundancy is summarized in Table 1.

**Table 1:** Different approaches to handle gene-set redundancy.

Tool	Gene-set redundancy correction method	Type of visualization support	Supports any gene-set source	Supports any enrichment test	Supports enrichment comparison
POSOC [21]	Modified gene-sets	None			
Ontologizer [22]	Modified test	None			
GOStats [23]	Modified gene-sets	None			
elim [23]	Modified gene-sets	None			
weight [24]	Modified test	None			
OntoExpress [25]	None	Hierarchical	♦		
BiNGO [27]	None	Hierarchical			
WebGestalt [28]	None	Hierarchical			
DAVID [12], [29], [30]	None	Table	♦		
MCM [31]	None	Network	♦		
ClueGO [32]	None	Network			
Enrichment Map	None	Network	♦	♦	♦

*Gene-set redundancy correction methods* typically utilize modified gene-sets (*Modified gene-sets*) or rely on a modified enrichment test (*Modified test*), but usually require hierarchically structured gene-sets; consequently, they usually do not support gene-sets from resources other than Gene Ontology. Other methods offer different *types of visualization support*, adopting a *hierarchical*, *tabular* or *network* organization of gene-sets. These methods usually allow a broader choice of gene-set sources and enrichment tests. The table also indicates whether the method supports comparison of different enrichment results.

To overcome the above limitations, we developed the Enrichment Map visualization method, which organizes gene-sets into a similarity network, where nodes represent gene-sets, links represent the overlap of member genes, and node color encodes the enrichment score. ClueGO and MCM create similar networks, however, Enrichment Map uses a visual style that we find more intuitive and offers improved functionality: two different enrichment experiments can be comparatively analyzed by displaying them in the same map; new query gene-sets (e.g. disease genes, targets of regulators) can be compared to existing gene-sets post-analysis; a heat-map can be used to explore the data underlying the enrichment results (e.g. gene-expression patterns) for any gene-set; finally, Enrichment Map is modular, enabling use with any type of enrichment test or gene-set source. Enrichment Map is implemented as a freely available and open-source plugin for the Cytoscape network visualization and analysis software [26].

We next describe how Enrichment Map works and how it can be used to interpret enrichment analysis results using frequently encountered experimental designs.

## Results

**Figure 1:**
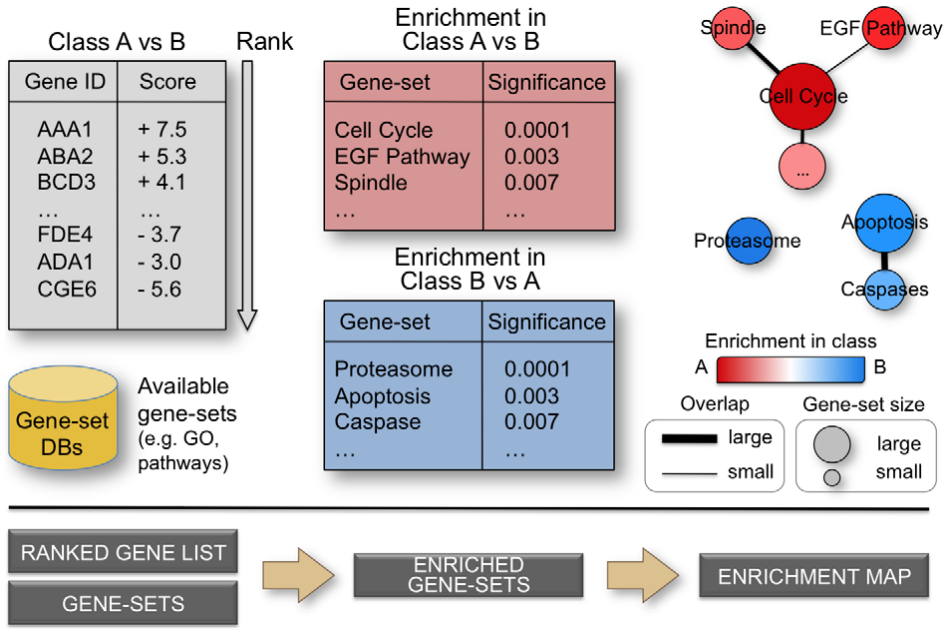
From ranked gene lists to the enrichment map. High-throughput genomic experiments often output large gene lists, which are typically ranked according to a statistic measuring difference in one experimental condition versus another. Ranked lists are analyzed for enrichment in known sets of functionally related genes (e.g. pathways) from publicly accessible databases. An enrichment map is drawn, representing the enrichment results as a network of gene-sets (nodes) related by their similarity (edges), with enrichment significance encoded by the node color gradient, where color intensity represents significance and color hue (red / blue) represents the class (i.e. biological condition) of interest. Node size represents the gene-set size and edge thickness represents the degree of overlap between two gene-sets.

To simplify the navigation and interpretation of enrichment results, we have developed Enrichment Map, a network-based gene-set enrichment result visualization method. Gene-sets are first analyzed for enrichment significance using a method of choice, e.g. GSEA [13], and then organized as a weighted similarity network, where nodes represent gene-sets and weighted links (i.e. edges) between the nodes represent an “overlap” score depending on the number of genes two gene-sets share. Nodes are automatically arranged so that highly similar gene-sets are placed close together; these clusters can be easily identified manually and related to biological functions. Gene-set enrichment results are graphically mapped to the Enrichment Map: node size represents the number of genes in the gene-set; edge thickness is proportional to the overlap between gene-sets, calculated using the Jaccard or overlap coefficients (see Methods). The enrichment score (specifically, the enrichment p-value) is mapped to the node color as a color gradient. In a typical enrichment test for a single set of genes (one-class), node color ranges from white (no enrichment) to red (high enrichment). In a two-class experiment design, node color ranges from red (high enrichment in one class e.g. case) to white (no enrichment) to blue (high enrichment in the second class e.g. control). In the specific case of a gene expression experiment where a condition of interest is compared to a baseline control, red is interpreted as up-regulation and blue as down-regulation. Figure 1 summarizes the information flow from gene scoring to Enrichment Map analysis for a typical, two-class experiment.

In the next sections, we give examples of typical analysis scenarios where Enrichment Map is used to analyze gene-expression experiments. For simplicity, only Gene Ontology derived gene-sets are used, although any gene-sets can be used in practice. *Use case 1* presents the most basic application of Enrichment Map, the analysis of a two-class experiment. We analyze a gene expression data set of MCF7 breast cancer cells, in presence or absence of estrogen treatment at 24 hours of culture. In *Use case 2*, we compare the estrogen response at two time-points, 12 and 24 hours, to evaluate changes over time. In *Use case 3*, we analyze a gene expression study of colon cancer and use the query set feature of Enrichment Map to investigate the relationship between the gene expression signature and known genes associated with colon cancer.

### Use case 1: One enrichment (estrogen treatment of breast cancer cells)

Here, we analyzed the changes in gene expression associated with estrogen treatment of a breast cancer cell line (MCF7) at 24 hours of culture [33]. Enrichment results were generated after scoring genes for differential expression using the *t-test* statistic, comparing the estrogen-treated versus the untreated samples. GSEA was then used to find enriched GO gene-sets in up- or down-regulated genes. Only gene-sets passing conservative significance thresholds (p-value<0.001, False Discovery Rate (FDR)<5%) were selected for display in the Enrichment Map, resulting in 156 total gene-sets (out of 2378) significantly enriched in treated (148 gene-sets) or untreated cells (8).

The output of GSEA, like many other enrichment methods, consists of a table of gene-sets and their enrichment statistics. This organization is not helpful for enrichment interpretation if too many gene-sets pass the significance threshold, as is the case here. Although the table can be ranked according to enrichment significance (nominal p-value, FDR, or other scores, in the case of GSEA), it is difficult to identify gene-sets belonging to a common functional group, because they are typically scattered throughout the table. To demonstrate this problem, multiple microtubule cytoskeleton-related gene-sets are highlighted in the enrichment table for estrogen-treated cells (Table S1, first tab).

**Figure 2:**
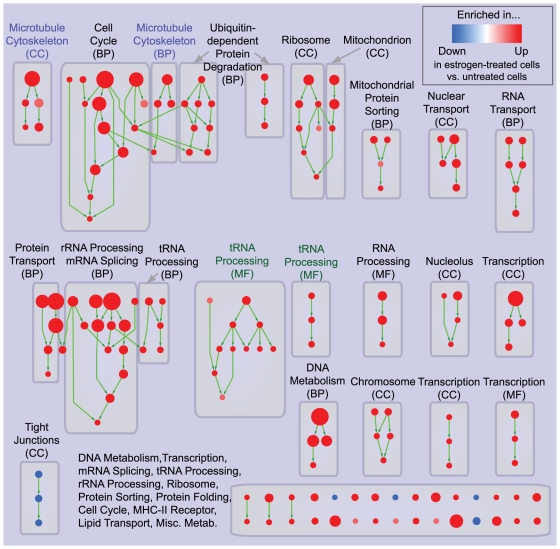
Hierarchical visualization of enrichment results for estrogen treatment of breast cancer cells. Hierarchical organization of GO gene-set enrichment results for estrogen-treated compared to untreated breast cancer cells at 24 hours of culture. Nodes represent gene-sets and edges represent GO defined relations (*Is-a*, *Part-of*, *Regulates*). Gene-sets that did not pass the enrichment significance threshold are not shown. Nodes are colored according to enrichment results: red represents enrichment in estrogen-treated cells (i.e. up-regulation after estrogen treatment), whereas blue represents enrichment in untreated cells (i.e. down-regulation after estrogen treatment). Color intensity is proportional to enrichment significance. Since conservative thresholds were used to select gene-sets, most of the node colors are intense (corresponding to highly significant gene-sets). Subnetworks (i.e. clusters) are annotated according to the corresponding function. The acronym in brackets represents the specific GO ontology the gene-sets belong to: Molecular Function (MF), Cellular Component (CC), Biological Process (BP). Microtubule cytoskeleton (purple labels) and tRNA processing (green labels) were highlighted to show absence of connections between related gene-sets.

A simple approach to this problem consists of visualizing enriched GO gene-sets according to the hierarchical relations defined in the ontology (Figure 2). The resulting network is composed of several, disconnected sub-networks (i.e. clusters). These are not interconnected because gene-sets failing the enrichment significance threshold were removed to limit the network size, which otherwise would be too large for visualization purposes. Clusters typically map to one or a few functional groups, as displayed by the manually added annotation labels in Figure 2, hence they can be successfully used to summarize enrichment results. However, gene-sets relating to the same biological function (e.g. *Microtubule cytoskeleton*) but defined in different GO partitions (e.g. *Cellular Component* and *Biological Process*) are systematically split into different clusters. In a few cases, this occurs even for functionally related gene-sets from the same GO partition (e.g. *tRNA Processing* from *Molecular Function*).

**Figure 3:**
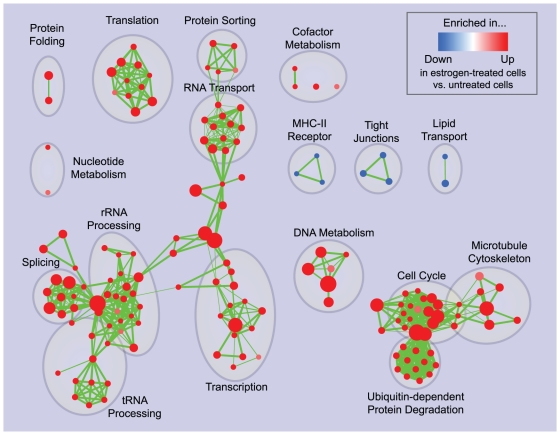
Enrichment map for estrogen treatment of breast cancer cells at 24 hours of culture. The map displays the enriched gene-sets in estrogen-treated vs. untreated breast cancer cells at 24 hours of culture. As in Figure 2, red node color represents enrichment in estrogen-treated cells (i.e. up-regulation after estrogen treatment), whereas blue represents enrichment in untreated cells (i.e. down-regulation after estrogen treatment); color intensity is proportional to enrichment significance. Clusters of functionally related gene-sets were manually circled and assigned a label.

**Figure 4:**
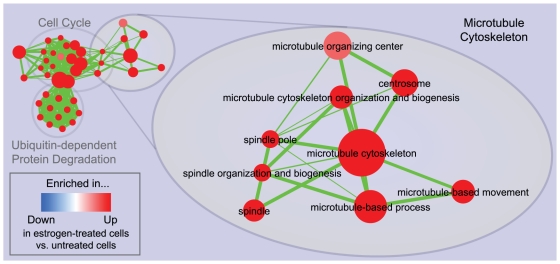
Zoom in of the microtubule cytoskeleton cluster in the 24 hours estrogen treatment enrichment map. Cytoskeleton-related gene-sets from different GO partitions, such as *Spindle* (*CC*) and *Microtubule-based process* (*BP*) have been grouped together, unlike in the purely hierarchical visualization in Figure 2. As in the previous figures, red node color represents enrichment in estrogen-treated cells (i.e. up-regulation after estrogen treatment), whereas blue represents enrichment in untreated cells (i.e. down-regulation after estrogen treatment); color intensity is proportional to enrichment significance.

The Enrichment Map for the same data displayed in Figure 3 overcomes these problems. Gene-sets are organized according to their mutual overlap. Minimal editing, such as minor repositioning of nodes and removal of few exceedingly generic gene-sets (e.g. *Protein Complex Assembly*, *Biopolymer Catabolism*), was done to optimize the map layout. Clusters were manually circled and labeled to highlight the prevalent biological functions among a set of related gene-sets. Functionally related gene-sets are highly connected, to a larger extent than in the purely hierarchical visualization, as exemplified in the case of *Microtubule cytoskeleton* (Figure 4). Most importantly, the overall functional “landscape” fits with the known role of estrogen hormones as activators of cell proliferation [34]. In fact, gene-sets enriched in estrogen-treated cells (in red) relate to increased protein synthesis and RNA processing (left side of the map) and to the execution and regulation of mitotic cell cycle (right side of the map). Gene-sets found enriched in untreated cells (blue) constitute a minor portion of the map and relate to membrane and cell adhesion (namely, MHC-II receptors, tight junctions and lipid transport). The down-regulation of these functions may have a role in supporting proliferation or it may be associated with relatively undifferentiated cellular states.

The approach described in this use-case can be applied to any genomic experiment generating a ranked list of genes. For example, genes can be ranked by their likelihood of being regulated by a given transcription factor, according to ChIP-chip or ChIP-seq experiments (*Chromatin Immunoprecipitation* of genomic DNA followed by chip hybridization or sequencing) and then GSEA, or any whole-distribution method, can be used to test gene-set enrichment in top-ranking genes.

### Use case 2: Two enrichments (estrogen treatment of breast cancer cells)

**Figure 5:**
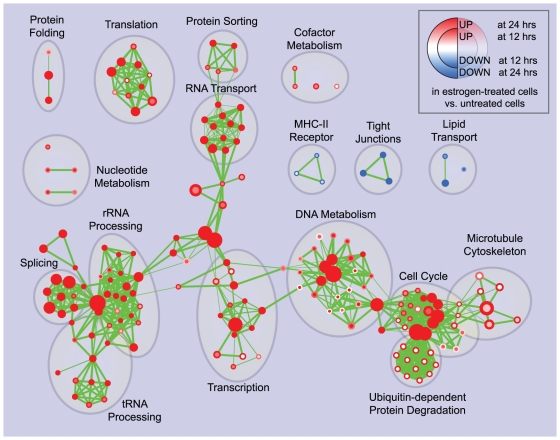
Enrichment map for estrogen treatment of breast cancer cells at 12 and 24 hours of culture. The map displays the enriched gene-sets in estrogen-treated vs. untreated breast cancer cells at 12 and 24 hours of culture. Enrichments were mapped to the inner node area and to the node borders, respectively. As in the previous figures, red represents enrichment in estrogen-treated cells (i.e. up-regulation after estrogen treatment), whereas blue represents enrichment in untreated cells (i.e. down-regulation after estrogen treatment); color intensity is proportional to enrichment significance. Clusters of functionally related gene-sets were manually circled and assigned a label.

Enrichment Map can be used to analyze experiments with more complex designs than the basic two-class described above. In the previous use case, gene expression was analyzed looking for changes associated with estrogen treatment at 24 hours of culture. Here we evaluate differences in the estrogen response kinetics by additionally considering gene expression enrichment at 12 hours of culture. Genes were scored for differential gene expression (*t-test*) by comparing treated and untreated cells at matching culture time-points (12 and 24 hours, respectively). GSEA was used to find enriched GO gene-sets in up- or down-regulated genes, as in the previous use case. Applying the same conservative significance thresholds (p-value<0.001, FDR<5%), 188 total gene-sets (out of 2378) were found significantly enriched in treated (179) or untreated cells (9). The enrichment map was generated by mapping the 12 hour enrichment to the node center and the 24 hour enrichment to the node border (Figure 5). This two-enrichment visualization is useful, as we can see gene-set groups that have the same (all red or all blue) or different enrichment across the two data sets. It is immediately apparent that the agreement between the 12 and 24 hours estrogen response is very high - most nodes are all one color and no nodes are both blue and red, which would indicate a gene-set with opposite enrichment in the two time points. In certain cases there are nodes that are significantly enriched at one time-point, but not at the other.

**Figure 6:**
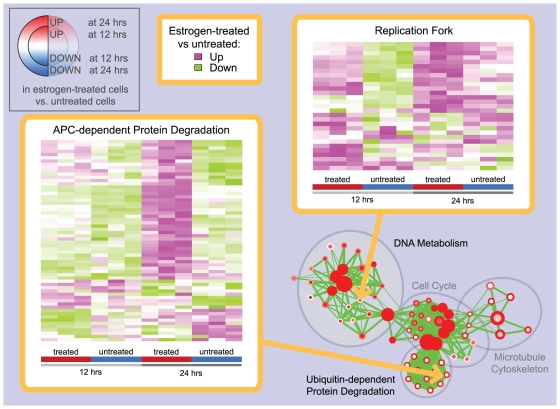
Heat-maps displaying gene-set expression patterns in the estrogen treatment experiment. Two gene-sets displaying different enrichment patterns at 12 and 24 hours of the estrogen treatment experiment were selected from the enrichment map in Figure 5 and their expression patterns were explored using heat maps within the Enrichment Map software. For *APC-dependent protein degradation* (left), there is a marked increase of gene expression in estrogen treated cells at 24 hours compared to 12 hours, whereas the gene levels for untreated cells are substantially the same at the two time points; the pattern observed is consistent with the presence of significant enrichment only at 24 hours. On the other hand, for *Replication fork* (right), gene expression in estrogen treated cells at 12 and 24 hours is globally at similar levels, whereas there is an increase of gene levels in untreated cells. This suggests an explanation of why *Replication fork* is enriched only at 12 hours.

Gene-sets with stronger enrichment at 24 compared to 12 hours are present in most of the functional groups. These results suggest that the transcriptional response to estrogen treatment is globally stronger at 24 hours and that the functional groups induced or repressed are essentially the same. Further, the four clusters relating to DNA metabolism, Cell cycle, Microtubule cytoskeleton and Ubiquitin-dependent protein degradation present an interesting pattern: gene-sets relating to DNA synthesis (such as *Replication fork*, *DNA polymerase activity*) are characterized by stronger enrichment at 12 hours, whereas gene-sets relating to G2/M phase components and processes (such as *Chromosome condensation*, *Spindle* and *Anaphase Promoting Complex (APC)-dependent protein degradation*) prevail at 24 hours. First, we investigated if differences in enrichment significance at 12 and 24 hours are consistent with differential gene expression patterns, utilizing heat-maps generated by the Enrichment Map software. The *APC-dependent protein degradation* (GO:0031145, full name: Anaphase-promoting complex-dependent proteasomal ubiquitin-dependent protein catabolic process) gene-set is characterized by a markedly stronger induction after estrogen treatment at 24 compared to 12 hours (Figure 6, left pane), which is consistent with enrichment results. We next investigated a gene-set exhibiting the opposite enrichment pattern, *Replication Fork* (GO:0005657) (Figure 6, right pane). Gene expression in estrogen-treated cells is moderately higher at 24 than 12 hours, but there is a comparable increase in the untreated cell expression levels. Since enrichments were computed by comparing estrogen treated and untreated cells at the same time-point, the different enrichment observed for DNA metabolism gene-sets is likely due to the estrogen-independent increase of gene expression levels in untreated cells. This suggests that G2/M phase execution could be more dependent on estrogen signaling than G1 phase, at least for MCF7 breast cancer cells.

To evaluate the effect of alternative statistics for measuring differential gene expression on enrichment results, we repeated the analysis using the *ratio of class means* instead of the *t-test* statistic. Although more gene-sets were found enriched, the enrichment map clusters were globally the same, but characterized by noisier patterns (Text S1). For this reason, the ratio of class means was not used for the final analysis. This also demonstrates the utility of Enrichment Map in guiding the choice of parameters and statistical tests for enrichment analysis.

### Use case 3: query set post-analysis (early onset colon cancer)

**Figure 7:**
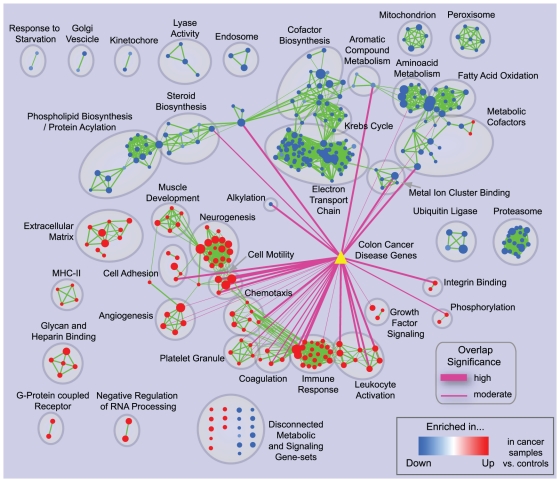
Enrichment map for early-onset colon cancer and overlap with known disease genes. The map displays the enriched gene-sets in early onset colon cancer patients vs. normal controls. The yellow triangle represents the set of known colon cancer genes from the DiseaseHub database, which integrates disease gene lists from several genotype-phenotype association resources. Purple edges indicate overlap between the disease signature and enriched gene-sets; thickness represents significance. Only edges with a Fisher's Exact Test nominal p-value smaller than 10^−4^ were visualized.

Here, we analyze the gene expression profiles of early-onset colon cancer mucosa versus control samples [35] to identify functional groups that are enriched in differential gene expression. We then mine these gene-sets for differentially expressed genes that have known disease associations, or that may be new disease gene candidates, using the *query set post-analysis* feature of the Enrichment Map software. Gene expression data were scored for differentiality between cases and controls using the *t-test* statistic; GSEA was used to generate enrichment results, which were then visualized using Enrichment Map, following the procedure described in *Use case 1*. Known colon cancer genes were obtained from the DiseaseHub database (http://zldev.ccbr.utoronto.ca/~ddong/diseaseHub/), which integrates data from OMIM (Online Mendelian Inheritance in Man), GAD (Genetic Association Database), HGMD (Human Gene Mutation Database), PharmGKB (Pharmacogenomics Knowledge Base), CGP (Cancer Genome Project) and GWAS (Genome Wide Association Studies). Most of the disease genes in this database harbor rare mutations or polymorphisms linked to colon cancer either by causation or by statistical association. Overlap was scored using *Fisher's Exact Test* p-value. Nominal p-values (i.e. not adjusted) smaller than 10^−4^ were deemed significant, and visualized as pink edges with thickness indicating significance level (proportional to −log (p-value)) (Figure 7).

Overall, the enrichment map has a smaller number of up-regulated than down-regulated gene-sets (125 and 234, respectively). Many down-regulated gene-sets map to metabolic processes, with clusters relating to functions such as phospholipid and steroid biosynthesis, metabolic cofactors, amino acid metabolism and oxidative metabolism (specifically, citric acid cycle, mitochondrion, electron transport chain). This is not surprising, as it is well known that cancer cells undergo major metabolic shifts, such as the *Warburg Effect*, consisting of dramatically reduced oxidative metabolism and mitochondrial activity [36]. Compared to up-regulated gene-sets, however, only a few down-regulated sets significantly overlap with known disease genes. This is reasonable since the metabolic switch observed in cancer is likely a downstream consequence of neoplastic transformation, neither specific for colon cancer nor having a causal role in its development. We decided to evaluate the gene expression patterns of the three metabolic gene-sets with the most significant connections to known disease genes: *Iron ion binding* (GO:0005506), p-value 5.3×10^−13^, in the *Metal ion cluster binding cluster*, *Alkyl/aryl transferase activity* (GO:0016765, full name: Transferase activity, transferring alkyl or aryl (other than methyl) groups) p-value 8.2×10^−11^, in the *Alkylation* cluster, and *Electron carrier activity* (GO:0009055), p-value 1.3×10^−10^, in the *Metabolic cofactor* cluster. Disease genes from *Iron ion binding* and *Electron carrier activity* are highly overlapping (union size: 31, intersection size: 20), hence they were considered together. Most of these genes belong to the cytochrome P450 family (Table S2), which is important for xenobiotic metabolism. Mutations in this gene family have been associated with cancer either because of the impaired capability to neutralize toxic chemicals, or because of the acquired capability to activate the toxicity of otherwise inert compounds [37]. However, none of the cytochrome P450 genes is characterized by marked up-regulation or down-regulation (Table S2). On the other hand, *NQO1* (*NAD(P)H dehydrogenase*, *quinone 1*; EntrezGene ID: 1728), whose inactivating mutation has been associated with colon cancer in animal models and in human population studies [38], is markedly down-regulated in cancer samples (*t-test* nominal p-value  =  2.4×10^−6^, 66.5% average reduction compared to control). Although this gene is likely important for cancer progression and severity, it was not identified in the original published gene expression analysis. Inspection of *Alkyl/aryl transferase activity* revealed that more than 90% of the disease genes in this set are glutathione S-transferases, involved in detoxification. Specifically, *MGST1* (*Microsomal glutathione S-transferase 1*; EntrezGene ID: 4257) is markedly down-regulated (*t-test* nominal p-value  =  6.9×10^−5^, 65.0% average reduction compared to control) and certain *MGST1* polymorphisms have been associated with increased colon cancer risk [39]. Like *NQO1*, this gene was not identified in the original gene expression analysis. Similar results were also found for another metabolic gene-set, *Aromatic compound metabolic process* (GO:0006725), where the most down-regulated disease genes are *glucuronosyltransferases*. All in all, this type of analysis was useful to dissect the down-regulation of specific detoxification enzymes from the broader signal of down-regulation of oxidative metabolism (*Warburg Effect*). The biological relevance of the genes identified was supported by two independent sources, namely differential expression in the microarray experiment analyzed and known disease associations based on genetic screening or mechanistic studies. Therefore these genes likely play an important role in colon cancer, and should be considered for further study.

The up-regulated gene-sets with the highest overlap with known disease genes are related to adhesion, angiogenesis, cell motility and immune response. Among these, we focused on the gene-sets *Cell Motility* and *Chemotaxis*. Dysregulation of these processes is likely to be responsible for the tumor invasive growth, its infiltration into the lymph nodes in more advanced stages and eventually metastasis [40], [41]. We first looked at the *Cell Motility* gene-set (GO:0051270), which is characterized by a very significant overlap with the disease gene-set (nominal p-value: 3.3×10^−8^). However, the disease genes in this set are only weakly or inconsistently up-regulated (most significant nominal p-value: 1.8×10^−3^). On the other hand, the *Chemotaxis* gene-set (GO:0006935) has several known disease genes that are also significantly up-regulated, even if its overall overlap significance (2.5×10^−5^) is weaker than for *Cell Motility*. Most of these genes are chemokine ligands (Table S2), among which the most up-regulated is *CXCL12* (*Chemokine (C-X-C motif) ligand 12 (stromal cell-derived factor 1*), EntrezGene ID: 6387). *CLXC12* is secreted by carcinoma-associated fibroblasts, and is considered responsible for tumor invasion [41]. Since this gene-set is likely important for colon cancer, we also looked for differentially expressed genes that were *not* in our disease gene-set. Interestingly, the top-ranking gene in this group is *CYR61* (*Cysteine-rich*, *angiogenic inducer*, *61*; EntrezGene ID: 3491), which was identified in the original study as one of the seven genes with the most consistent differential expression between colon cancer and control samples [35]. *CYR61* could be a good new candidate for disease association, as only two publications relating *CYR61* to colon cancer were found in PubMed [35], [42], one being the original publication of the microarray data set used in this analysis. This shows how the *query set post-analysis* feature of the Enrichment Map software can be used to identify gene-sets associated with a biological condition according to independent data sources (in this case, differential gene expression and known disease genes), and then to mine these sets for previously uncharacterized genes exhibiting interesting patterns. The applicability of this feature is not restricted to disease genes: it can also be used to identify the relations between the targets of a known regulator (e.g. transcription factor, microRNA) and the functional groups enriched in the condition(s) of interest.

### Enrichment Map can be applied to any enrichment test or gene set database

The previous use cases demonstrate the utility of Enrichment Map to visualize GSEA enrichment results, using Gene Ontology as gene-set source. However, Enrichment Map is compatible with any type of enrichment test or gene-set source. In Text S2 we show how it can be applied to a disease gene list, using *Fisher's Exact Test* to test a larger collection of gene-sets derived from Gene Ontology as well as pathway databases. We also compare Enrichment Map visualization with other available tools that are strictly dependent on *Fisher's Exact Test*.

### Limitations of Enrichment Map

Enrichment map works well when enrichment results contain many related gene sets. If only a few gene-sets result from enrichment analysis, Enrichment Map does not provide much benefit for result interpretation, as it is relatively easy to scan a list of a few gene-sets. Further, if the resulting gene-sets are not highly related, as they may be when not using Gene Ontology derived or similarly hierarchically organized gene-sets, Enrichment Map will not show clusters and thus will provide little benefit over a table of gene-sets presentation format. In Text S3 we evaluate the performance of Enrichment Map for several gene-set sources, including experimentally- or computationally-derived gene-sets, showing that Enrichment Map can be productively used in many cases, even when Gene Ontology is not the major gene-set source. However, specific gene-set sources, such as curated signatures from gene expression experiments in Text S3, may have sparser overlaps resulting in poorly connected networks.

## Discussion

We have described Enrichment Map, a method for gene-set enrichment visualization. Enrichment Map organizes enriched gene-sets in a network in a way that helps manage the large overlap between gene-sets that often complicates interpretation of gene-set enrichment results. Highly redundant or biologically related gene-sets are placed close together, making enrichment results easier to interpret. Gene-sets can be linked by various criteria, such as the amount of co-expression of member genes, however the gene-set overlap measure that we use has the advantage of being intuitive, as biologically similar sets are clustered together, and general, as it does not depend on the type of data being analyzed (e.g. gene expression or genetic associations). We have demonstrated the utility of the enrichment map visualization in analyzing two published microarray gene expression experiments, profiling the estrogen response in breast cancer cells and early onset colon cancer. We showed that Enrichment Map provides a concise and biologically meaningful view of the cellular processes and components characterized by differential expression.

Gene-set enrichment is often used to analyze single experiments, or single comparisons between two conditions within multi-condition experiments, and meta-analysis of gene expression data are often performed using Venn diagrams or heat-maps (for instance, [33], [43], [44]), without exploiting the full potential of functional annotations. Enrichment Map can be used for more informative comparisons, identifying which functional groups differ between experiments. A visualization framework is essential for this, as traditional displays of enrichment require tedious and error prone navigation of flat tables, often resulting in investigators choosing only a handful of gene-sets for follow-up out of the thousands available to them in a genomics experiment. The heat map view in Enrichment Map enables the user to zoom in and explore an enriched gene-set in more detail and the query set analysis facilitates exploration of relations to known disease genes or regulatory modules.

Gene-set enrichment has been successfully applied to link functional gene-sets to disease and other biological conditions in hundreds of publications (according to *ISI Web of Knowledge*, October 2010, DAVID and GSEA were quoted by 159 and 1421 papers, respectively). We have successfully applied Enrichment Map to several research projects such as cardiac failure [45], thyroid cancer signaling (Borrello MG, Degl'Innocenti D, Gariboldi M, Merico D, Antoniotti M, Pierotti M, Unpublished work) and autism [46]. Virtually any research project in genomics can take advantage of this visualization framework; in particular, the modular design of the Enrichment Map Cytoscape plugin software makes it easy to integrate within existing analysis workflows.

Future work will include incorporating molecular interaction network and pathway information [47], [48] into gene-set analysis methods, as has successfully been done for gene expression analysis [49], [50], [51], [52], [53]. We will also improve the visualization. For instance, we are working on methods to automatically summarize gene-set clusters using tag clouds (Oesper L, Merico D, Isserlin R, Bader GD, Submitted work) and on better visualization methods for multi-condition experiments (more than two enrichment results). It will also be useful to develop gene set similarity measures weighted to consider the most informative genes in the gene set (such as the most differentially expressed).

## Materials and Methods

### Microarray data analysis

All microarray gene expression data were downloaded from the NCBI GEO (Gene Expression Omnibus) database. The raw .CEL files were processed with the *rma* statistical model for gene expression signals, using the Bioconductor [54] *affy* package. The data-sets were selected according to the following quality criteria: reliable and high-coverage microarray platform (Affymetrix HGU-133 plus 2.0), clear experimental design, sufficient number of replicates (≥3 for cell lines, ≥5 for patient samples), uniform cell composition, *Principal Component Analysis* (*PCA*) results compatible with the experimental design (i.e. clear separation of samples from different classes). Enrichment analysis was performed after conversion from Affymetrix to NCBI Entrez-Gene identifiers, utilizing the Bioconductor *hgu133plus2* package (downloaded March 2009).

#### Estrogen treatment of breast cancer cells

The microarray data (GSE11352) were originally composed of 18 samples, with 3 replicates for every one of the 6 classes (3 time-points for treated and untreated). The subset composed of the 12 and 24 hour time-points was analyzed using GSEA, *t-test*, 2000 *gene-set* permutations. The enrichment maps in use case 1 and 2 were generated using only the gene-sets satisfying these enrichment thresholds: nominal p-value<0.001, FDR<5%. The enrichment map overlap coefficient was set to 0.5.

#### Early Onset Colon Cancer

The microarray data (GSE4107) are composed of 22 samples, with 10 normal and 12 colon cancer samples (colonic mucosa surgical samples). The data set was analyzed using GSEA, *t-test*, 2000 *gene-set* permutations. The enrichment map was generated using only the gene-sets passing the following thresholds: nominal p-value<0.001, FDR<5%. The overlap coefficient was set to 0.5.

### Gene-set pre-processing

Human Gene Ontology (GO) [11] annotations were downloaded from Bioconductor, *org.Hs.eg.db* package (March 2009). In order to maximize the coverage of GO annotations, no evidence code based filter was applied. Terms annotating more than 500 or less than 10 genes were discarded, resulting in 2378 GO terms being used for the analysis. These thresholds are routinely used in enrichment analysis as large gene-sets rarely convey much useful biological meaning (e.g. *regulation of physiological process*), whereas very small gene-sets are more susceptible to being falsely enriched due to random fluctuations. Also, the reduced number of gene-sets decreases the multiple testing correction burden, potentially increasing the power of the analysis.

### Enrichment Map: overlap measures and network visualization

Gene-set definition and enrichment table files are loaded in the Enrichment Map Cytoscape plugin and filtered for significance, according to the p-value and FDR thresholds set by the user. Overlap between significant gene-sets is computed according to the Jaccard coefficient or overlap coefficient, depending on the user's choice.

Given sets *A* and *B*, and the cardinality operator *| |* where *|X|* equals to the number of elements within set *X*, the *Jaccard coefficient (JC)* is defined as:
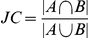
Whereas the *overlap coefficient (OC)* is defined as:
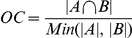
The overlap coefficient is better when hierarchically-organized gene-set collections (such as Gene Ontology) are included in the analysis. Parent-child overlap produces the maximal score (1), which means that all hierarchical relations will be present in the network. On the other hand, the Jaccard coefficient tends to group gene-sets with similar size, hence Gene Ontology parent-child relations are often absent from the network. The gene-set network is generated using only those interactions that pass a user-defined threshold for the Overlap or Jaccard coefficient and it is arranged using the Cytoscape *force directed* layout, *weighted* mode. The Overlap or Jaccard coefficient defines the edge weights in this case.

### Enrichment Map: implementation

Enriched Map was implemented as a Java plugin for the freely available Cytoscape network visualization and analysis software [26]. The plugin together with the source code is freely available at http://baderlab.org/Software/EnrichmentMap under the *GNU LGPL* license. The plugin reads two types of input formats, GSEA-specific and generic. Heat-map visualization, as described in *Use case 2*, is available for any selected gene-set. Any gene-set (or collection of gene-sets) of user's choice can be uploaded to perform the query set post-analysis, as described in *Use case 3*.

## Supporting Information

Table S1 GSEA enrichment results for estrogen treatment of breast cancer cells; gene-sets relating to the microtubule cytoskeleton are highlighted.
(0.68 MB XLS)

Table S2 Gene expression and annotation tables for genes analyzed in Use case 3 (post-analysis); genes discussed in the main text are highlighted.
(0.11 MB XLS)

Text S1 Enrichment Maps for estrogen treatment using different statistics for differential expression; ratio-of-class-means generates noisier results than the t-test.
(1.72 MB DOC)

Text S2 Enrichment Map for Alzheimer disease genes using Fisher's Exact Test and comparison to MCM.
(1.98 MB DOC)

Text S3 Enrichment Map analysis using additional gene-set sources.
(4.75 MB DOC)
