# Supplementary material for: Enrichment Map: A Network-Based Method for Gene-Set Enrichment Visualization and Interpretation
Source: PLoS One. 2010 Nov 15;5(11):e13984. doi: 10.1371/journal.pone.0013984 (PMC2981572; doi:10.1371/journal.pone.0013984)

**Supplementary File 2:**

**Evaluation of different statistics for differential expression.**

To evaluate the impact of the gene expression differentiality statistics, GSEA enrichment analysis was performed after ranking genes according to the ratio of class means (RCM). Enrichment results were globally similar (data not shown). The detailed inspection of clusters, as displayed below, revealed that RCM generates noisier results (bottom panel) than the t-test based ranking (top panel). For instance, several large gene-sets are enriched at 12 but not 24 hours (*DNA metabolic process*, *Chromosomal part*, *Cell cycle process*); however, the other nodes in their neighborhood are not characterized by the same enrichment pattern. This problem does not occur with t-test based ranking.


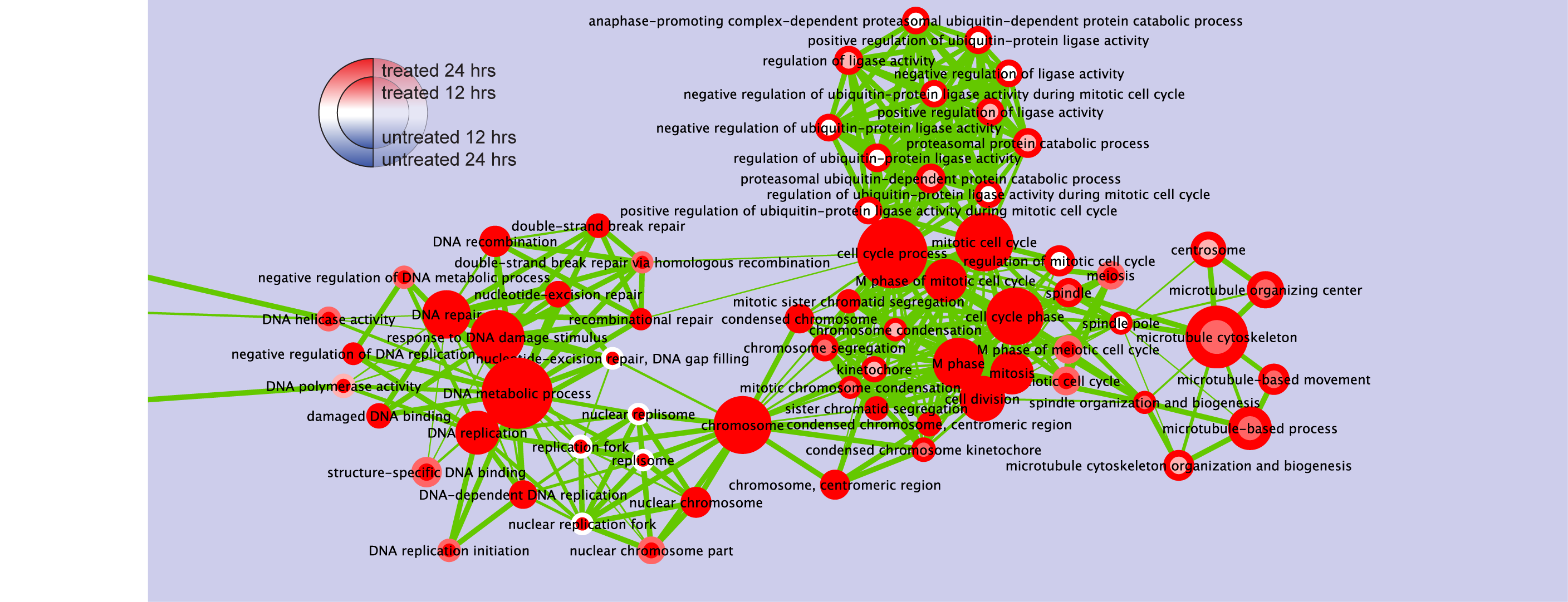


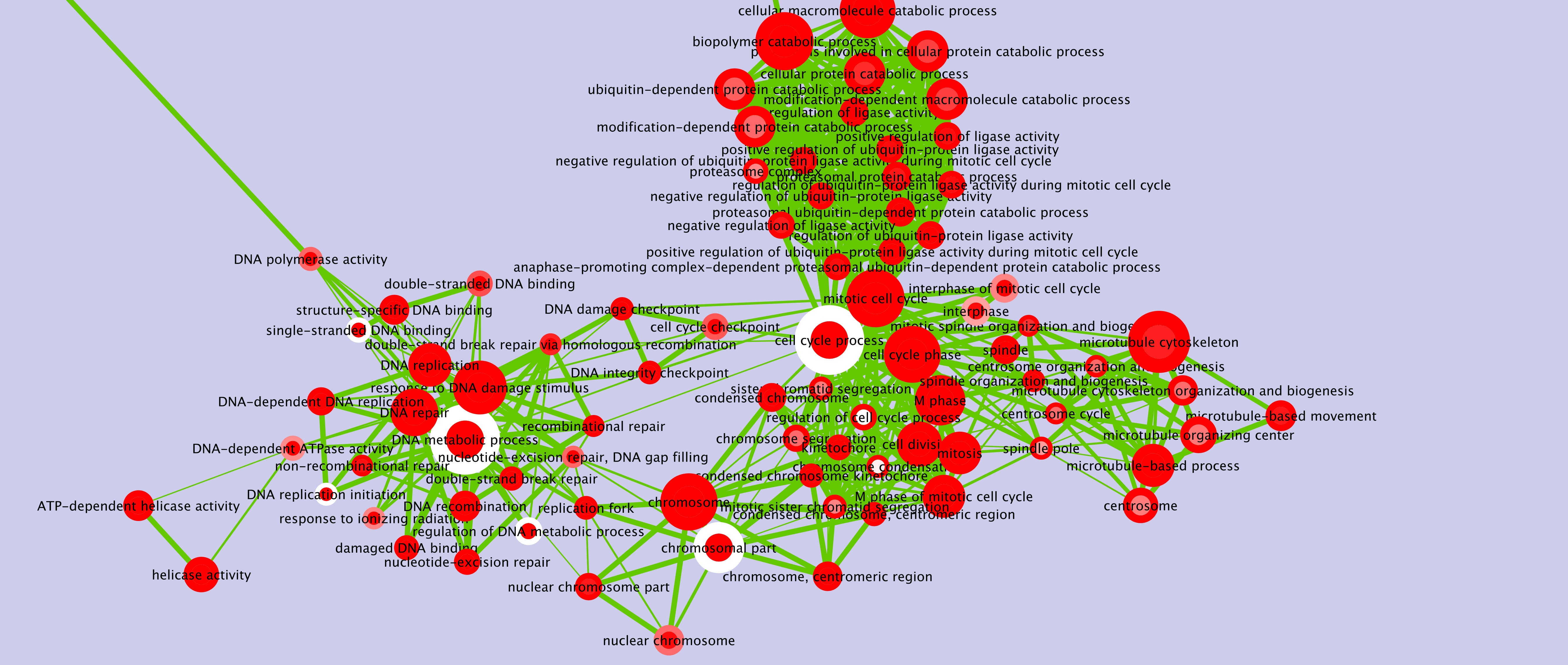

Supplement: Text S1 — Enrichment Maps for estrogen treatment using different statistics for differential expression; ratio-of-class-means generates noisier results than the t-test. (1.72 MB DOC) [file pone.0013984.s003.doc]
