# Supplementary material for: Enrichment Map: A Network-Based Method for Gene-Set Enrichment Visualization and Interpretation
Source: PLoS One. 2010 Nov 15;5(11):e13984. doi: 10.1371/journal.pone.0013984 (PMC2981572; doi:10.1371/journal.pone.0013984)
Supplement: Text S2 — Enrichment Map for Alzheimer disease genes using Fisher's Exact Test and comparison to MCM. (1.98 MB DOC) [file pone.0013984.s004.doc]

**Supplementary File 4**

**Enrichment analysis of Alzheimer Disease genes using Fisher’s Exact Test: comparison of Enrichment Map and Molecular Concept Maps visualization results**

**Outline**

Here we show the application of Enrichment Map to Fisher’s Exact Test enrichment results of Alzheimer’s Disease-associated genes identified by Blalock et al. (PNAS, 2004). The collection of tested gene-sets was extended by adding KEGG, NCI and Biocarta pathways to Gene Ontology, in order to show that substantial overlap exists among pathway-derived gene-sets as well.

To establish a comparison of Enrichment Map with other tools, the same experimental gene-sets was analyzed using MCM (Molecular Concept Maps), which uses Fisher’s Exact Test but is not compatible with other tests such as GSEA.

**Methods**

**Experimental data**

Blalock et al. (PNAS, 2004) tested transcript levels from healthy and diseased patients for correlation to quantitative pathological indexes. The two experimental sets of genes characterized by significant positive and negative correlation are stored in MSigDB as ALZHEIMERS_DISEASE_UP (1473 genes) and ALZHEIMERS_DISEASE_DN (1121 genes).

**Pathway gene-sets**

KEGG genes-sets were generated using the Bioconductor *org.Hs.eg.db* and *KEGG.db* packages (downloaded in March 2009). NCI gene-sets were downloaded from the NCI Pathway web-site (November 2009) and converted from Uniprot to Entrez-Gene ID using the Bioconductor *org.Hs.eg.db* package (downloaded in March 2009). Biocarta pathways were downloaded from WhichGenes (http://www.whichgenes.org/) in March 2010.

**Enrichment analysis**

Gene Ontology and pathway gene-sets were tested for enrichment in the experimental sets using a one-tail Fisher’s Exact Test. Gene-sets were then assigned to either experimental set (Alzheimer-up or Alzheimer-down) by evaluating which test produced the smaller p-value. The FDR q-value was empirically estimated separately for gene-sets enriched in Alzheimer-up and –down by resampling: 2000 randomly-sampled sets were generated, the number of genes equal to Alzheimer-up and -down respectively; these random sets were then tested for enrichment in Gene Ontology and pathway sets. This procedure offers the advantage of considering the highly correlated structure of the tested gene-set collection. In fact, the estimated FDR q-value are lower than Benjamini-Hochberg FDR, a correction procedure that assumes independence among tests. The Fisher’s Exact Test and FDR estimation were coded in R.

**Enrichment Map analysis**

Gene-sets with p-value ≤ 0.001 and FDR q-value ≤ 0.05 were selected for the Enrichment Map. The overlap coefficient threshold was set to 0.4. Unlike GSEA enrichments, the FDR q-value was used for node coloring. The Fisher’s Exact Test p-value was not used for node coloring; its linear form would not be suitable, as the p-values span many orders of magnitude; the *– log (p-value)* would not be suitable either, as it is not possible to a priori define an upper bound.

**Results**

**Enrichment map**

As usual, enrichment in up-regulation was represented by red and enrichment in downregulation by blue. Overall, the Enrichment Map (fig. 1) is similar to the maps generated in use-cases 1-3: overlapping gene-sets form clusters, which were manually labeled. Notably, of 46 enriched pathways, 37 are part of labeled clusters (80.4%). Although this fraction is lower than the one observed for Gene Ontology-derived gene-sets (94%), it is still high enough to argue for extensive overlap among non-GO pathway gene sets. Two clusters (*Oxidative metabolism*, *Cell cycle and proteasome*) are particularly crowded, and their structure cannot be satisfactorily resolved using the default overlap-weighted force-directed Cytoscape layout. For this reason, the corresponding subnetworks were laid out using the organic layout (purple boxes), significantly improving their readability.

The identified networks are consistent with the known biology of Alzheimer disease (Blalock et al., PNAS, 2004): oxidative metabolism is down-regulated, as it is often the case in stress conditions; neural transmission is downregulated, in agreement with loss of neuronal function; aopoptosis pathways are up-regulated, in agreement with neuron loss; the up-regulation of cell cycle, cell proliferation, cell motility and several pathways involved in growth signaling and adhesion in cancer likely reflects a compensatory mechanism for the neuron loss and/or the expansion of glial populations to cope with the tissue loss; lymphoid organ development up-regulation probably reflects an inflammatory response that is either the trigger or downstream consequence of neuronal distress.

The presence of three KEGG pathways, *Alzheimer’s disease*, *Parkinson’s disease* and *Epithelial signaling in H. pilori infection* in the oxidative metabolism cluster are worth an explanation, as these pathways have apparently little relation with metabolism. Alzheimer and Parkinson pathways include a large number of dehydrogenases (42 out 176 and 136 total genes respectively) that are part of the *mitochondrial electron transport chain*; this explains why they both localize in the oxidative metabolism cluster, in proximity of a group of gene-sets related to the electron transport chain. Epithelial signaling in H. pilori infection includes a large number of proton transport-coupled ATPases, which explains its localization in proximity of gene-sets involved in ATP synthesis. Therefore, the enrichment of these pathways should be considered very cautiously, as metabolic rather than signaling genes may be uniquely responsible for the bulk of the enrichment significance. Pathway scoring methods based on pathway topology, as proposed by Draghici et al (Genomics, 2003) [8], may be able to overcome this confusion effect. We note, however, that this problem is not systematic. For instance, pathways involved in growth signaling and adhesion (8 members, including several KEGG cancer pathways) form a clear cluster of their own that is attached to functionally related clusters (*Wnt signaling* and *MAPK cascade*).


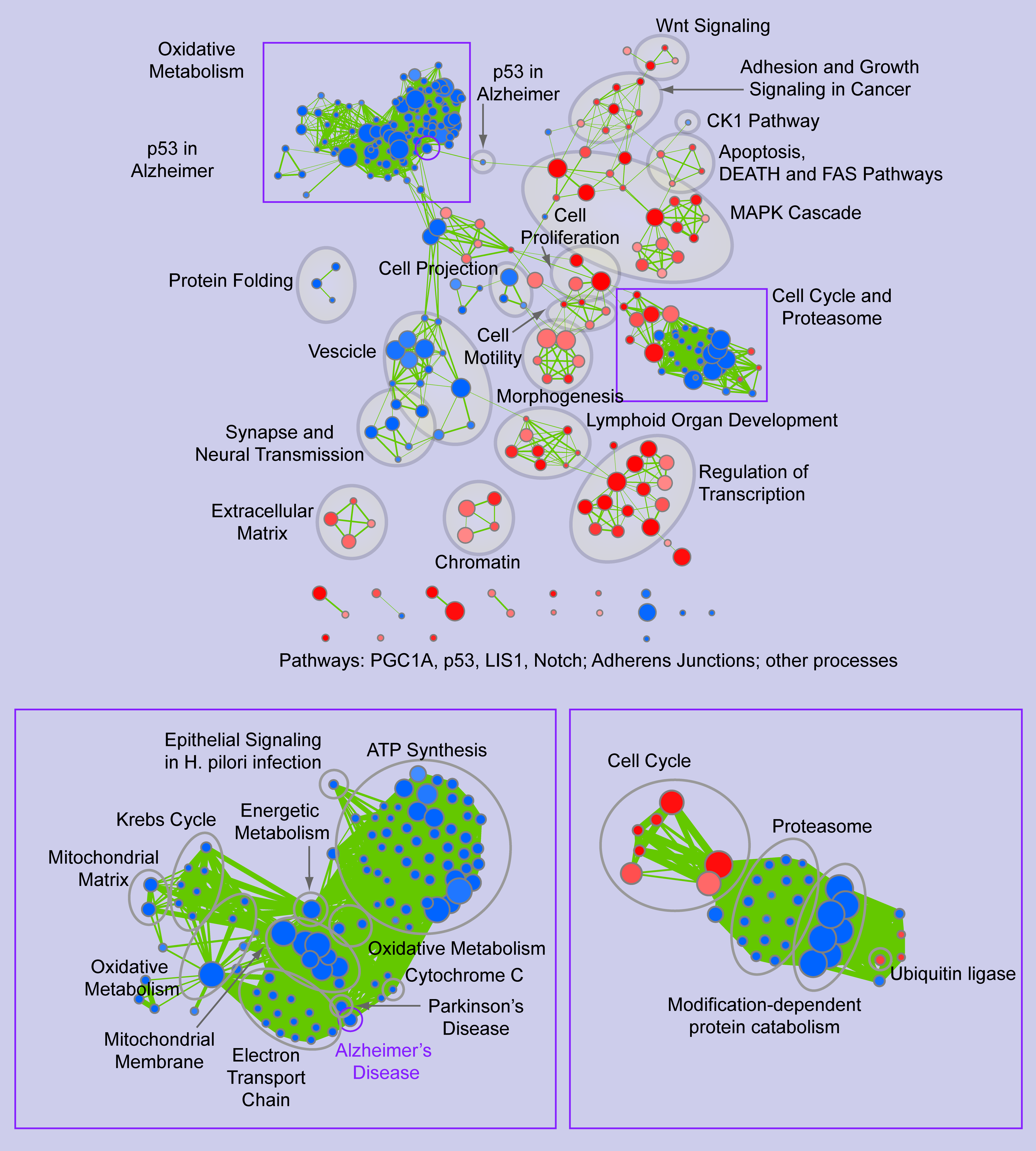


Figure 1. Enrichment Map for the experimental gene-sets positively (red) and negatively (blue) correlated to Alzheimer Disease. Two clusters, Oxidative metabolism and Cell cycle and proteasome (identified by purple boxes), were not well resolved by the Cytoscape force-directed layout, hence they were laid out as separate network using the organic layout. Specific subsets of functionally related gene-sets were manually identified and labeled.

**Molecular Concept Maps (MCM)**

The same experimental gene-sets (Alzheimer-up and -down) were submitted to MCM for analysis (http://private.molecularconcepts.org/main/index.jsp). MCM incorporates the same enrichment test (Fisher’s Exact Test) yet it applies specific gene-set filtering criteria; therefore, even after setting the same p-value threshold, it was not possible to reproduce the same enrichment results utilized as input for the Enrichment Map (Alzheimer-up: 42 gene-sets enriched in MCM and 109 in EM; Alzheimer-down: 46 gene-sets enriched in MCM and 170 in EM). In spite of this limitation, it is still possible to appreciate the main differences between the Enrichment Map and MCM visualization solutions.


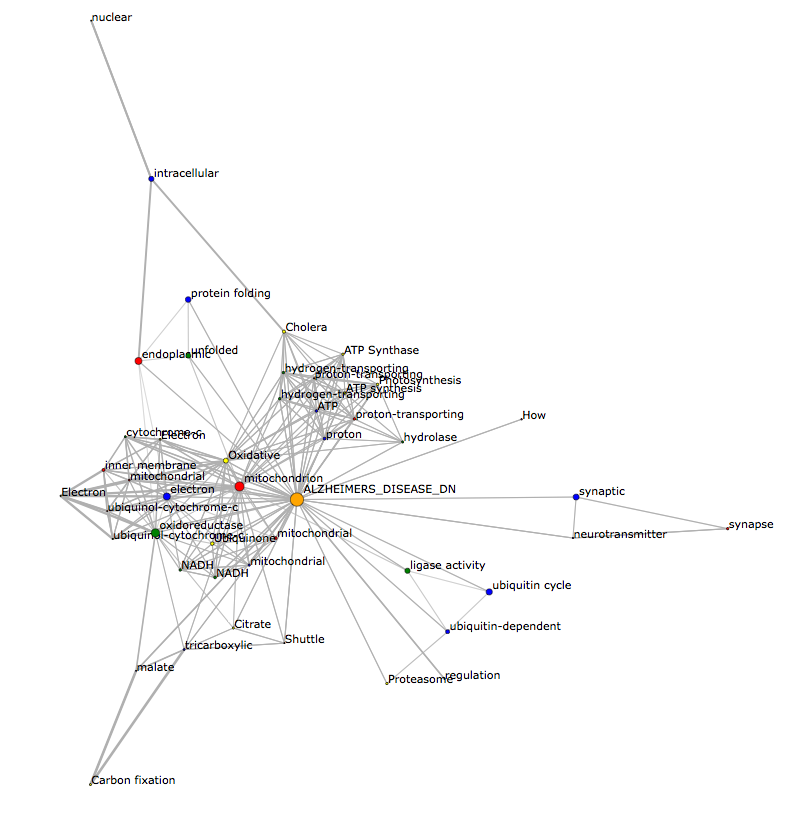


Figure 2. Molecular Concept Map for Alzheimer-down (full gene-set label length).

First of all, MCM does not let the user evaluate relations between gene-sets enriched in the up- and down-regulation sets, which are sometimes present as displayed by the Enrichment Map (e.g. cell cycle and proteasome cluster). Globally, in both MCM maps, clusters are generally less clearly delineated, which is likely due to the adoption of Fisher’s Exact Test p-value to weight set overlaps rather than the overlap coefficient used by Enrichment Map. The presence of different filtering criteria makes it hard to assess how MCM would fare with a number of nodes as large as 200-300, which is usually handled very well by Enrichment Map. Finally, the lack of support for functionalities such as node movement or display of selected nodes attributes makes it very difficult to interactively explore and interpret the results. The possibility to flip between short labels (fig. 2-3) and full-length labels (fig. 4-5) is not very helpful, as both views are affected by label overlap that is impossible to resolve given the static MCM images – ideally users would be able to manually move nodes around to optimize the layout.


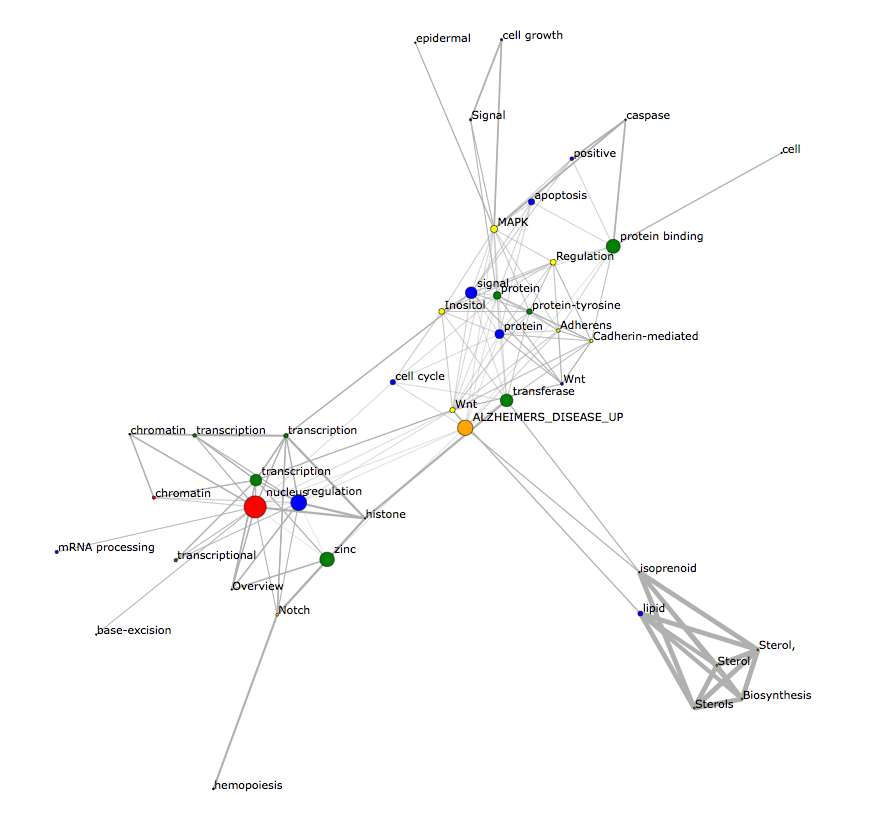


Figure 3. Molecular Concept Map for Alzheimer-up (limited gene-set label length).


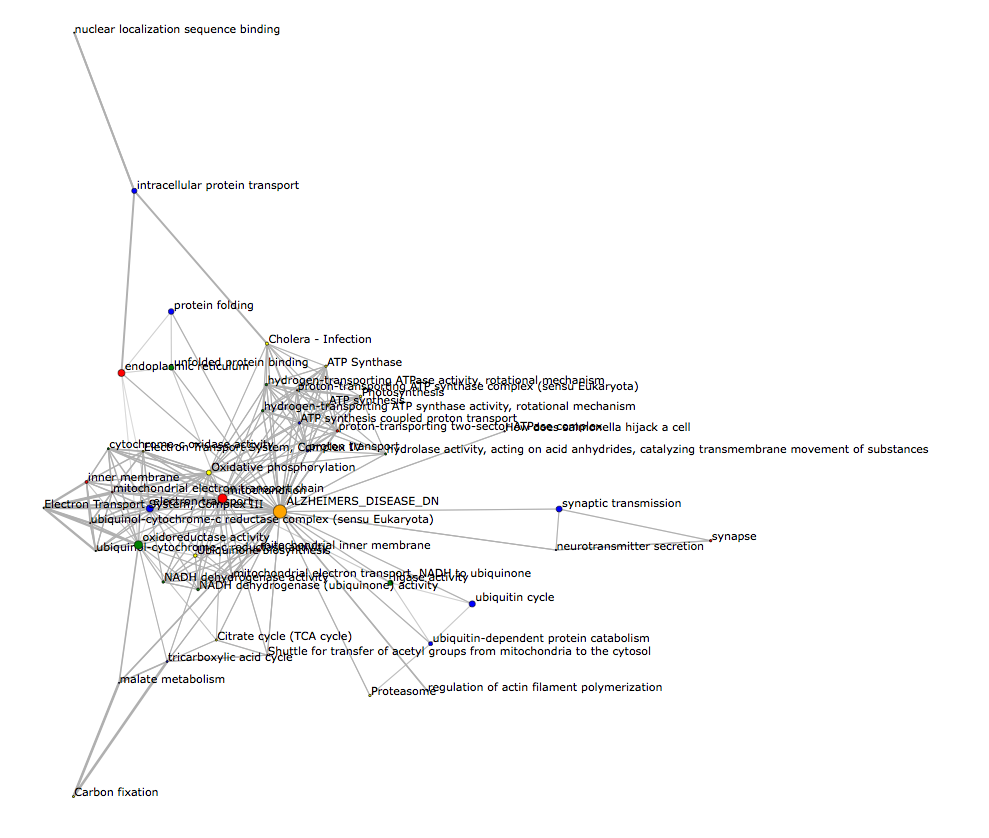


Figure 4. Molecular Concept Map for Alzheimer-down (full gene-set label length).


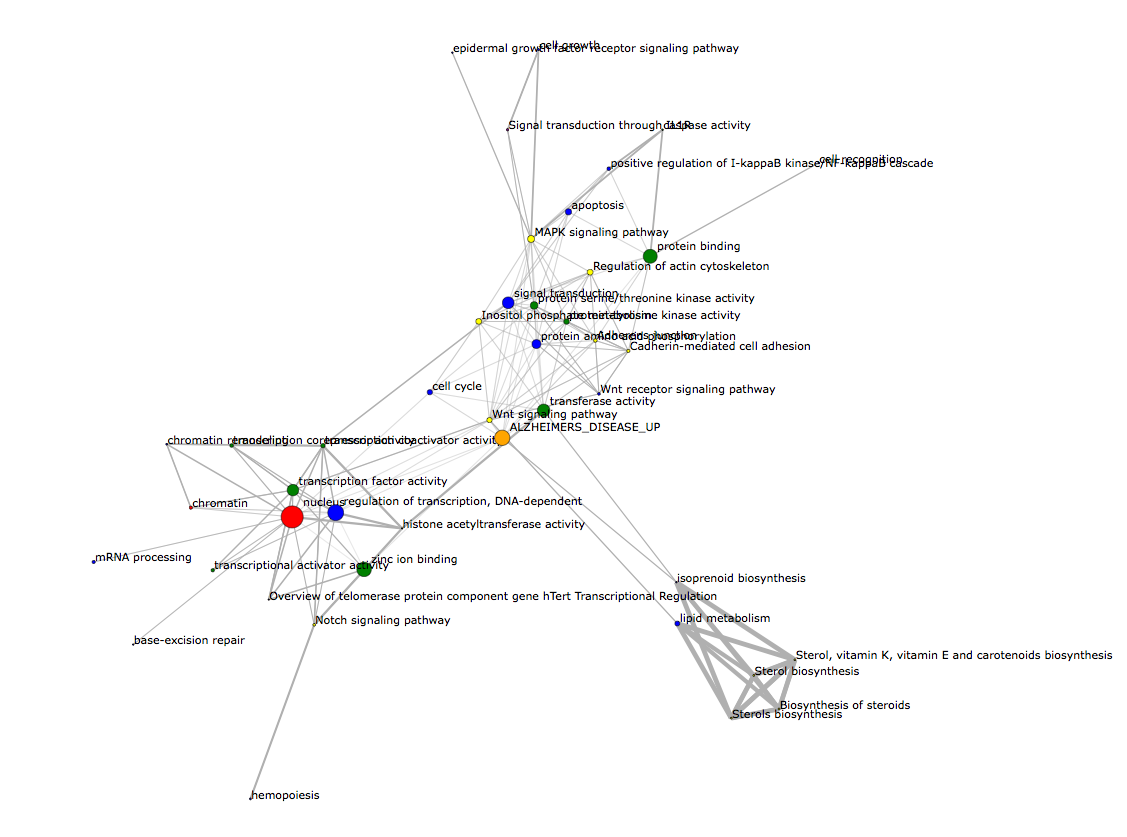


Figure 5. Molecular Concept Map for Alzheimer-up (limited gene-set label length).

**References**

Blalock EM, Geddes JW, Chen KC, Porter NM, Markesbery WR, Landfield PW.

Incipient Alzheimer's disease: microarray correlation analyses reveal major transcriptional and tumor suppressor responses.

Proc Natl Acad Sci U S A. 2004 Feb 17;101(7):2173-8.

PMID: 14769913
