# Supplementary material for: Enrichment Map: A Network-Based Method for Gene-Set Enrichment Visualization and Interpretation
Source: PLoS One. 2010 Nov 15;5(11):e13984. doi: 10.1371/journal.pone.0013984 (PMC2981572; doi:10.1371/journal.pone.0013984)
Supplement: Text S3 — Enrichment Map analysis using additional gene-set sources. (4.75 MB DOC) [file pone.0013984.s005.doc]

**Supplementary File 5**

**Enrichment Map analysis using additional gene-set sources**

**Outline**

To address the concern that using Gene Ontology (GO) is required to give Enrichment Map results displaying clear clusters, we analyzed gene sets without using GO. First, we analyzed the 12h time-point of breast cancer cells estrogen treatment, using gene-sets derived only from pathway databases (KEGG, NCI, Biocarta, Reactome). We then analyzed the transcriptional response of murine skin to UV radiation, using subsets of gene-sets available in MSigDB. Four different combinations of MSigDB gene-sets were used, to comprehensively evaluate the performance of Enrichment Map: 1) curated pathways (C2.CP), 2) curated pathways and computational cancer sets (C4), 3) pathways, computational cancer sets and curated gene expression signatures (C2.CGP), 4) pathways, computational cancer modules (C4.CM) and GO Biological Process (C5.BP). As can be seen below, enrichment maps that do not use hierarchically organized gene sets, such as GO, clearly display clusters.

**Methods**

**Estrogen treatment of breast cancer cells data**

Microarray data (GSE11352) were analyzed using GSEA, *t-test*, 2000 gene-set permutations. Gene-sets were derived only from pathway databases (KEGG, NCI, Biocarta, Reactome). KEGG genes-sets were generated using the Bioconductor *org.Hs.eg.db* and *KEGG.db* packages (downloaded in March 2009). NCI gene-sets were downloaded from the NCI Pathway web-site (November 2009) and converted from Uniprot to Entrez-Gene ID using the Bioconductor *org.Hs.eg.db* package (downloaded in March 2009). Reactome and Biocarta gene-sets were downloaded from *WhichGenes* (<http://www.whichgenes.org/>) in March 2010. Gene-sets with p-value ≤ 0.001 and FDR q-value ≤ 0.05 were selected for the Enrichment Map.

**Murine skin response to UV radiation**

Microarray data (GSE4066) were downloaded from NCBI GEO. The data were analyzed using GSEA, *t-test*, 2000 gene-set permutations. All gene-sets used for the enrichment analysis, including GO Biological Process, were downloaded from MSigDB (July 2010). Gene-sets with p-value ≤ 0.001 and FDR q-value ≤ 0.05 were selected for the Enrichment Map.

**Results**

**Analysis of estrogen treatment response (12h) using gene-sets from pathway databases (KEGG, NCI, Biocarta, Reactome)**

Enrichment is found only for up-regulated sets, similar to results described in *Use case 1-2*, where only Gene Ontology was used. The majority of the gene-sets belong to large clusters (i.e. > 10 nodes). *Cell cycle* and *DNA replication* related pathways form a large cluster (44/99 sets), connected to the smaller *Nucleotide metabolism* cluster. Most of the remaining gene-sets (44/99) form another large cluster, where multiple functions are present. We have identified specific sub-clusters for *Transcription*, *RNA transport* and *Glucose transport*, *tRNA modification*. A smaller cluster is formed by gene-sets related to *Protein folding*. Overall, most of the nodes belong to clusters with specific biological functions, although one of the clusters is more heterogeneous. Connectivity is also good, as only two pathways are not completely disconnected.


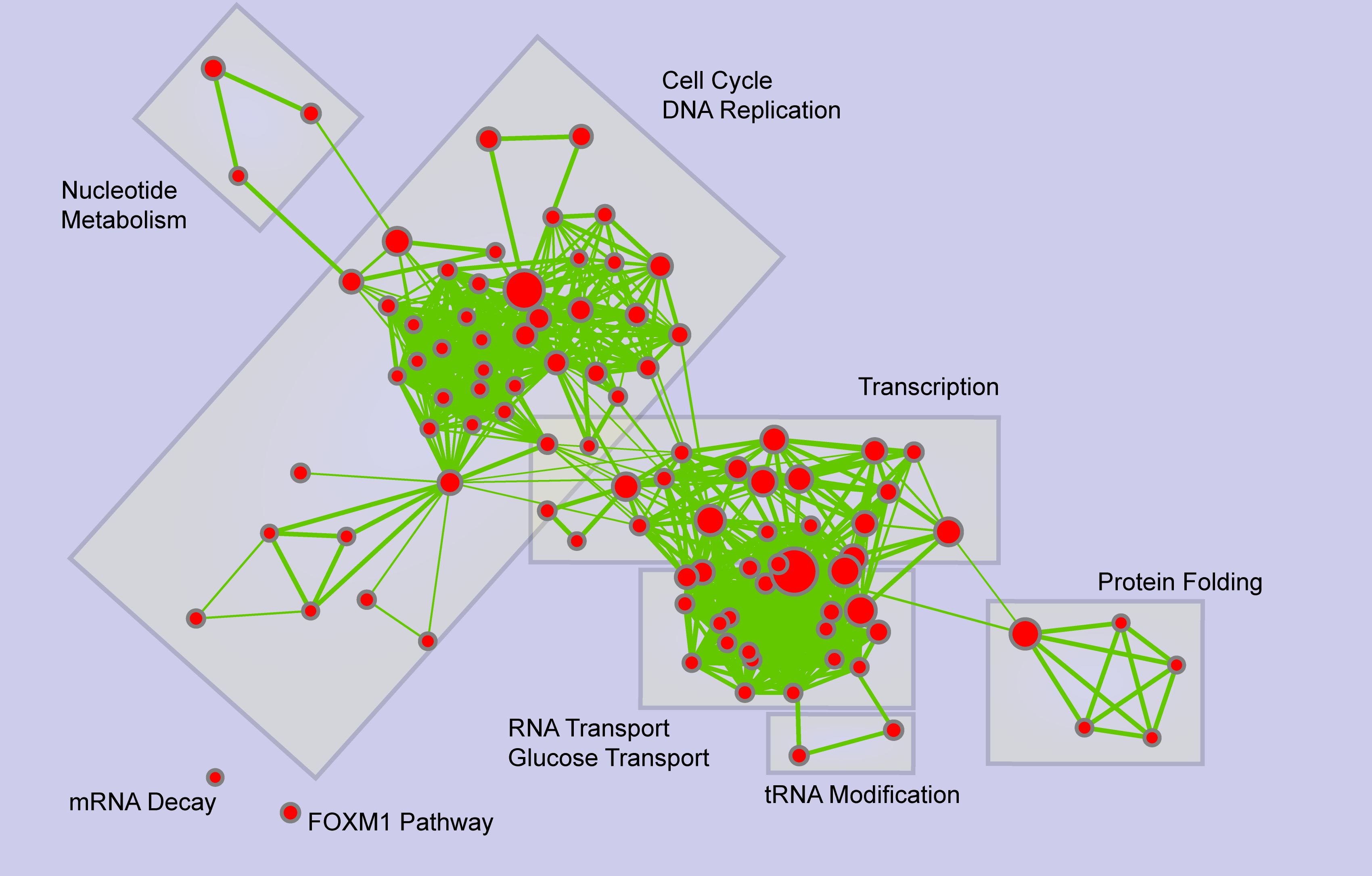


Figure 1. Enrichment map of 12h estrogen treatment of breast cancer cell. Only gene-sets from pathway databases were used.

This example shows that Enrichment Map can work well with gene-sets other than Gene Ontology. However, hierarchically-organized Reactome pathways (88/99) are prevalent in the map. Since this factor may be important for the network topology, in the next section we use Enrichment Map without any hierarchically organized gene-set source.

**Analysis of murine skin response to UV radiation using MSigDB gene-sets**

MSigDB pathways, computational modules and curated signatures do not have a pre-defined hierarchical structure. They were used to analyze the transcriptional response of murine skin to UV radiation.

**1) MSigDB pathways**

UV irradiation is known to activate DNA repair, apoptosis, inflammation and to inhibit cell growth. Most enriched pathways belong to these processes, confirming our expectations.

**
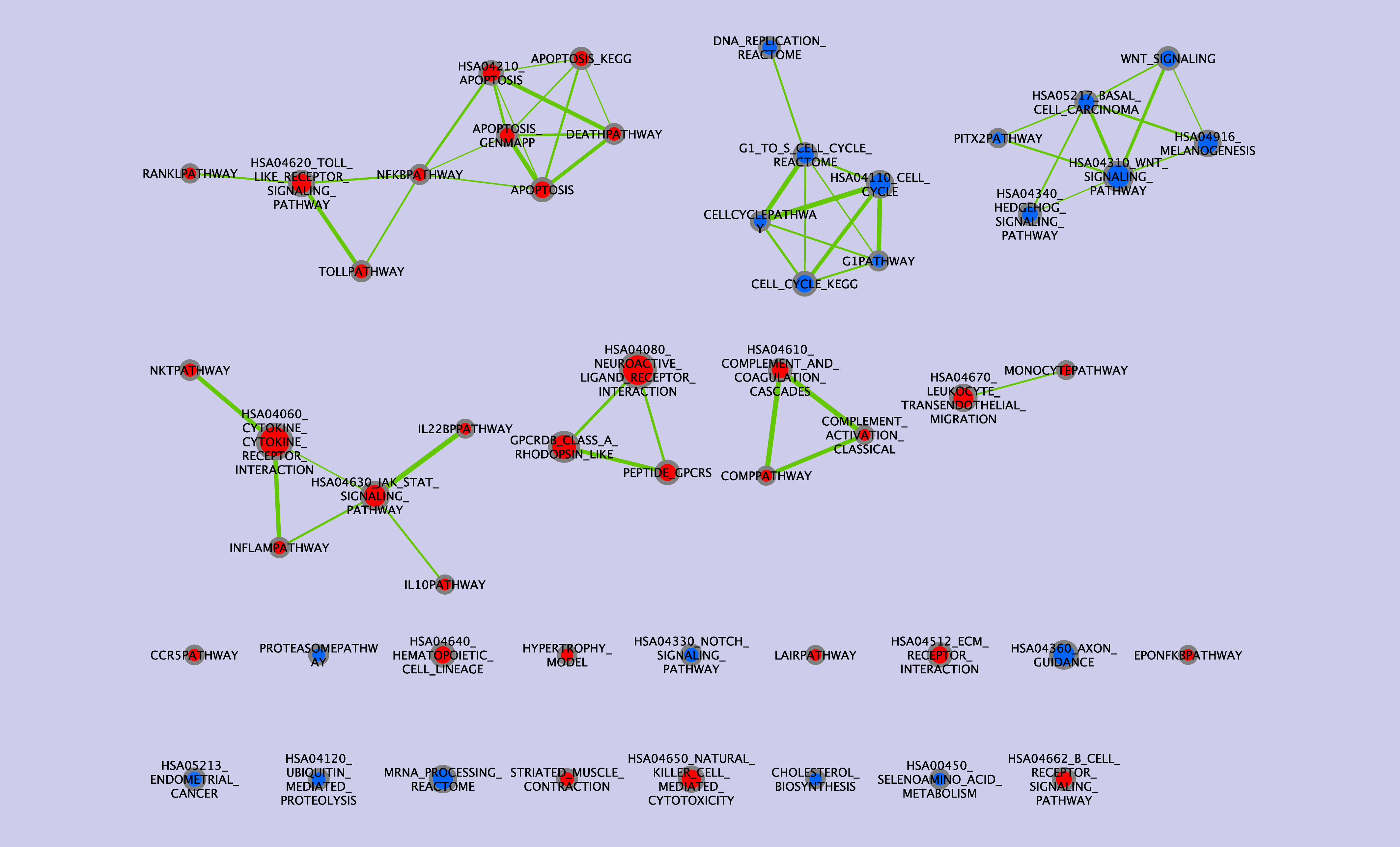
**

Figure 2. Enrichment map of murine skin response to UV radiation. Only gene-sets from MSigDB C2.CP (curated pathways) were used.

The majority of enriched gene-set (35/52) form small clusters (< 10 nodes); the other gene-sets (17/52) are disconnected from the rest of the network. Although pathways tend to be less connected than Gene Ontology gene-sets, as observed in Suppl. 4 (Text S4), Enrichment Map is useful to identify groups of pathways that are more redundant or biologically related.

**2) MSigDB pathways and computational cancer sets**

Computational cancer sets (C4) are strongly enriched in the response to UV irradiation: 321 (out of the 883 that were tested) are enriched in up- or down-regulation. Only a small fraction of the gene-sets are disconnected (28/373). The other gene-sets form small, independent clusters (71/373) or belong to the major connected component, which can be further divided into putative clusters (Figure 2, clusters not shown). Manual identification of biologically meaningful clusters is harder in this case because of gene-set annotation: C4.CM gene-sets (cancer modules, 130/373) are specifically hard to make sense of, as their names are not suggestive of any biological function. For this reason, in section (4) we analyze C4.CM together with MSigDB-derived Gene Ontology Biological Process.


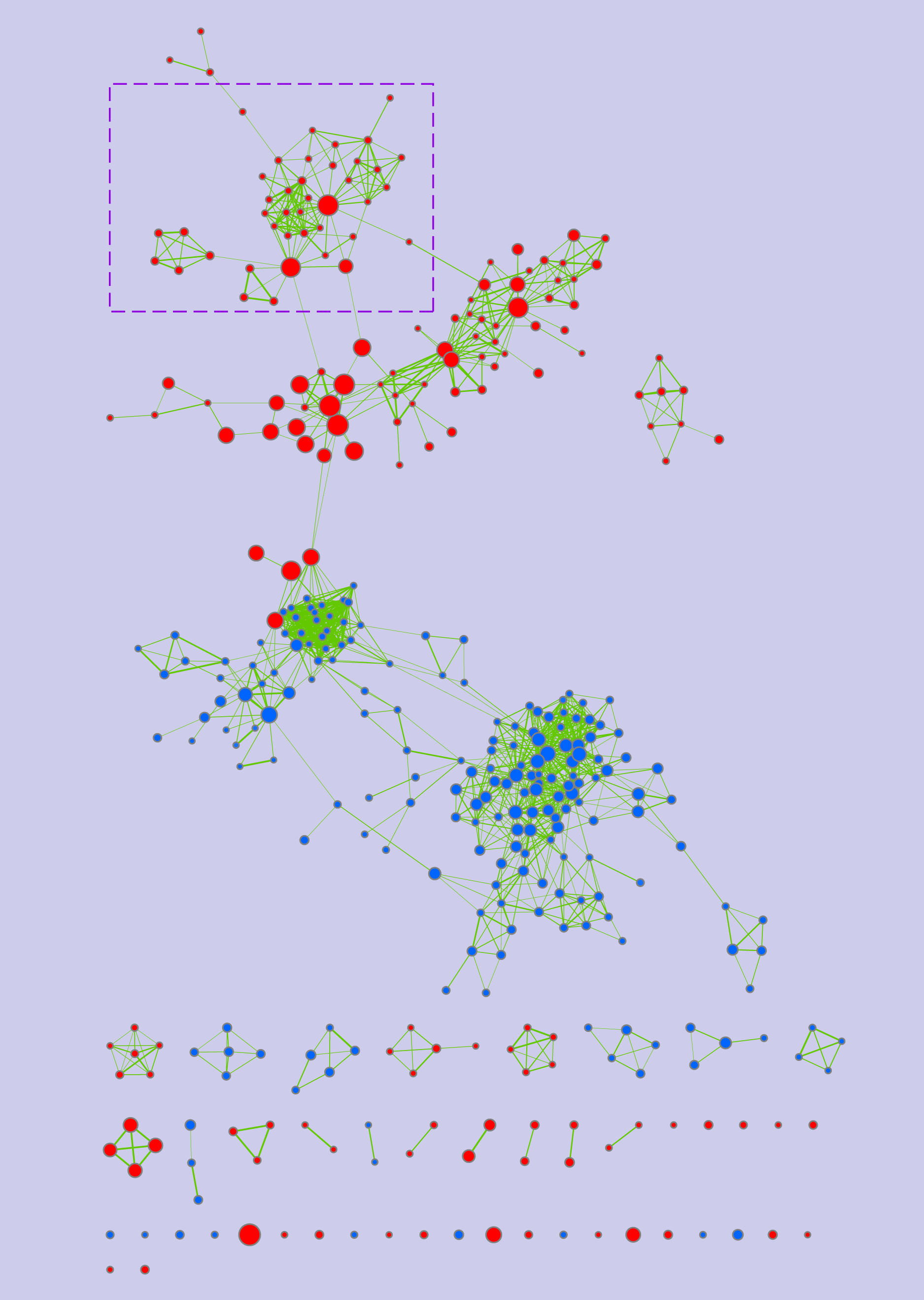


Figure 3. Enrichment map of murine skin response to UV radiation. MSigDB C2.CP (curated pathways) and C4 (computational cancer sets) were tested for enrichment. The sub-network delimited by the dashed purple line is displayed in detail in Figure 4.


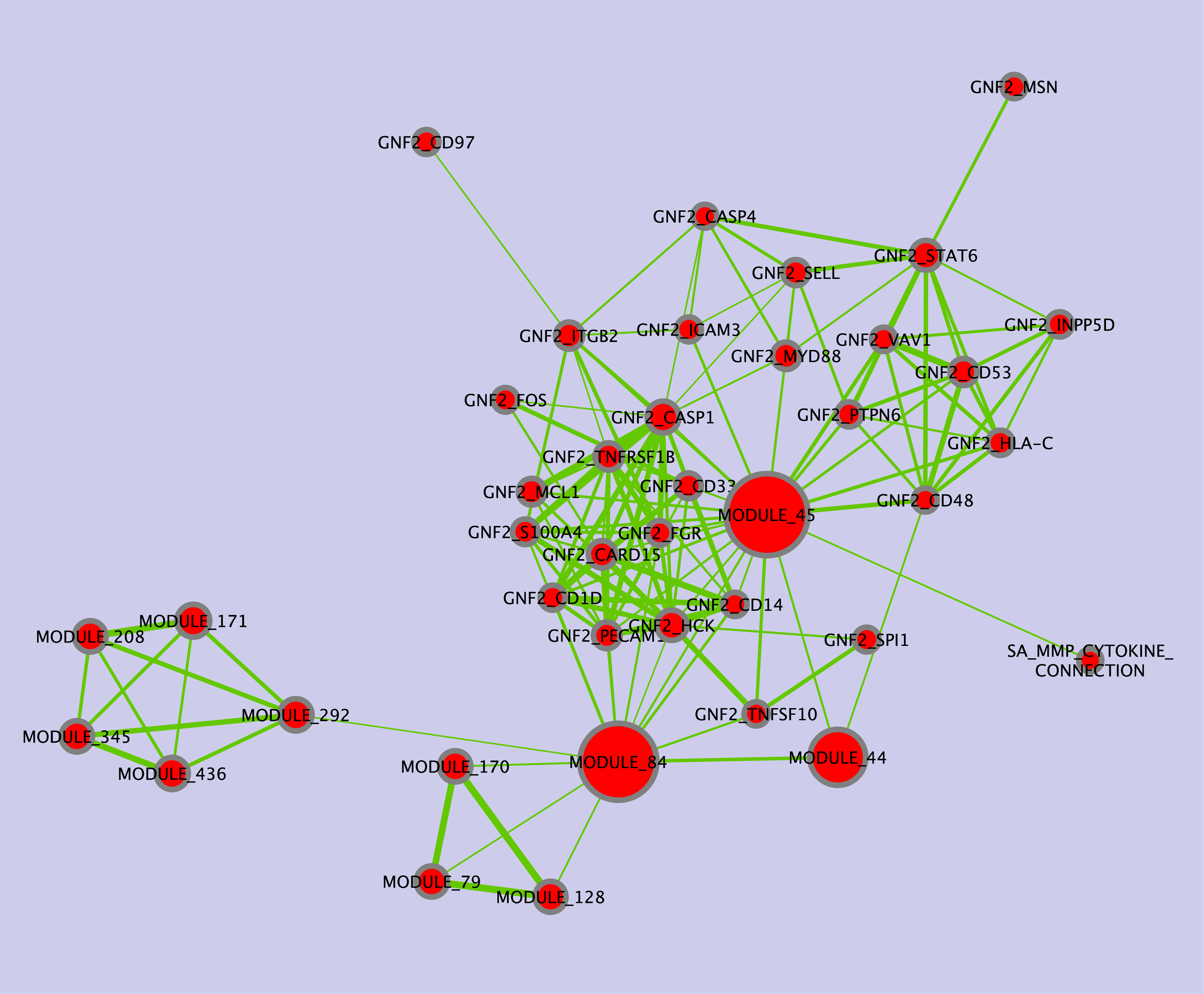


Figure 4. Putative cluster form the enrichment map in Figure 3 (murine skin response to UV radiation, MSigDB C2.CP and C4 gene-sets). C4.CM cancer sets are specifically hard to interpret, as they are labeled only using sequential numbers.

**3) MSigDB pathways, computational cancer sets and curated signatures**

Curated signatures (C2.CGP) display a tendency to be disconnected (140/277), even though many other gene-sets are present in the network. Consequently, the number and size of clusters does not change significantly after the addition of C2.CGP, whereas the number of singletons increases (Figure 5, bottom). Different explanations are possible. On one hand, this may reveal an intrinsic limitation of overlap as a means of organizing gene-sets: each signature may be composed of genes that belong to multiple functional groups, but their overlap to the other gene-sets is below the set overlap threshold. On the other hand, overlap between similar experiments may be small because only a handful of top-ranking genes (e.g. < 150) were selected in the curation process, resulting in limited reproducibility. Finally, it is also possible that every signature captures a unique and biologically meaningful set of genes, which is not present in other resources. Clusters were not labeled in this map for the same problem described in section (2) (the underlying gene sets have names that are hard to interpret). In conclusion, Enrichment Map does not perform well with MSigDB experimental signatures.


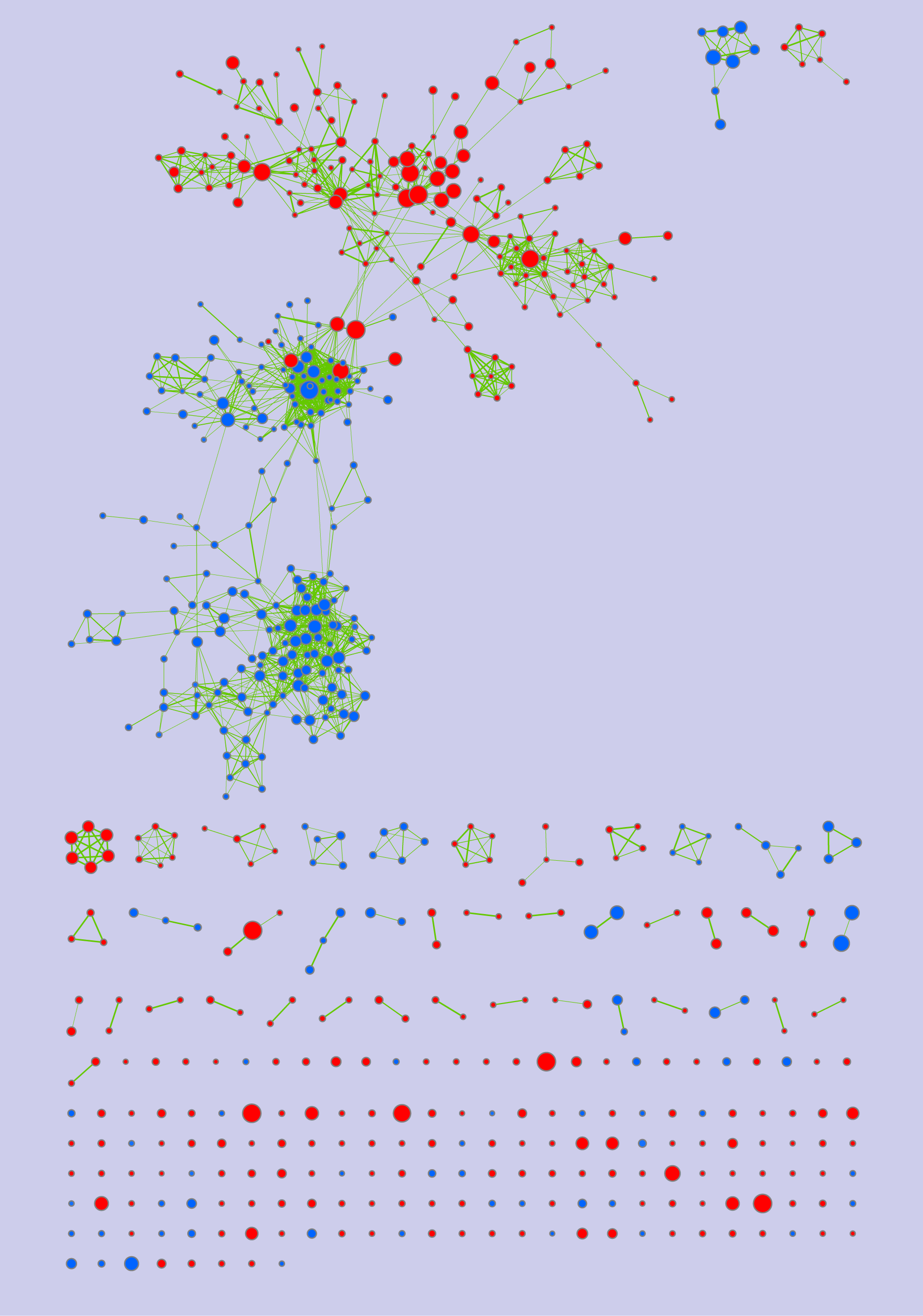


Figure 5. Enrichment map of murine skin response to UV radiation. MSigDB C2.CP (curated pathways), C4 (computational cancer sets) and C2.GCP (curated signatures) were tested for enrichment. Compared to Figure 3, and increased number of singletons can be noticed in the bottom part.

**4) MSigDB pathways, computational cancer modules and GO Biological Process**

This enrichment map is composed of 51 pathways, 130 cancer modules and 67 gene-sets derived from GO Biological Process (BP). Therefore Gene Ontology is not the dominant component, although its presence, together with pathways, can be exploited for cluster interpretation, as displayed in Figure 6. Only 27 out of 248 sets are not connected, whereas 207 form clusters of size 3 or larger. Several clusters can be identified within the major connected component (Figure 6). Overall, gene-sets display extensive overlap and clusters are usually meaningful. In many cases, Enrichment Map can be successfully used to put cancer modules in relation with gene-sets that have better annotations. However, some clusters are exclusively composed of cancer modules, hence it is not possible to evaluate their biological meaningfulness without using additional information that is not available in Enrichment Map.


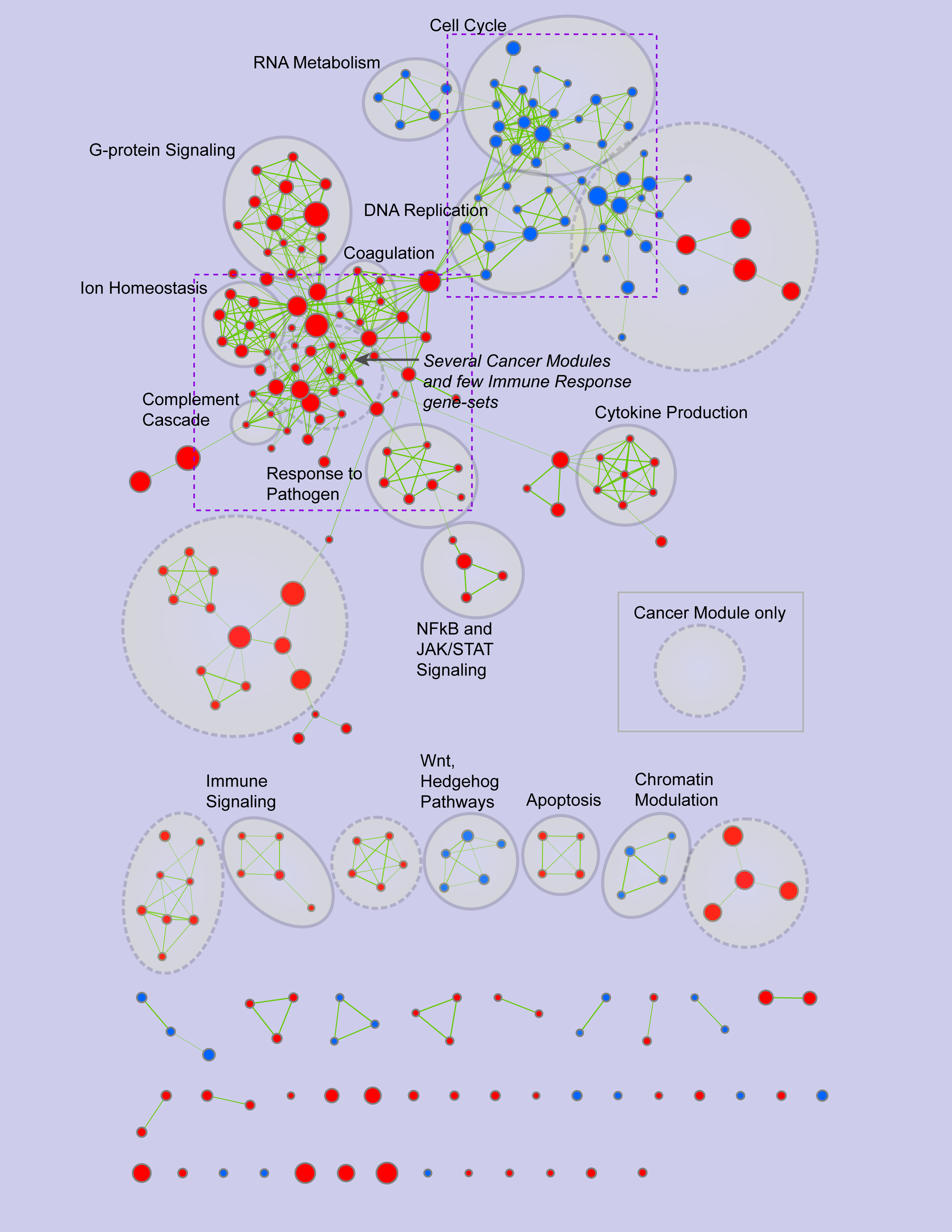


Figure 6. Enrichment map of murine skin response to UV radiation. MSigDB C2.CP (curated pathways), C4.CM (computational cancer modules) and GO Biological Process were used for the enrichment. Manually identified clusters that only have C4.CM-type gene-sets are circled by dashed lines and do not have labels. The sub-networks delimited by the dashed purple lines are displayed in detail in Figures 7 and 8.


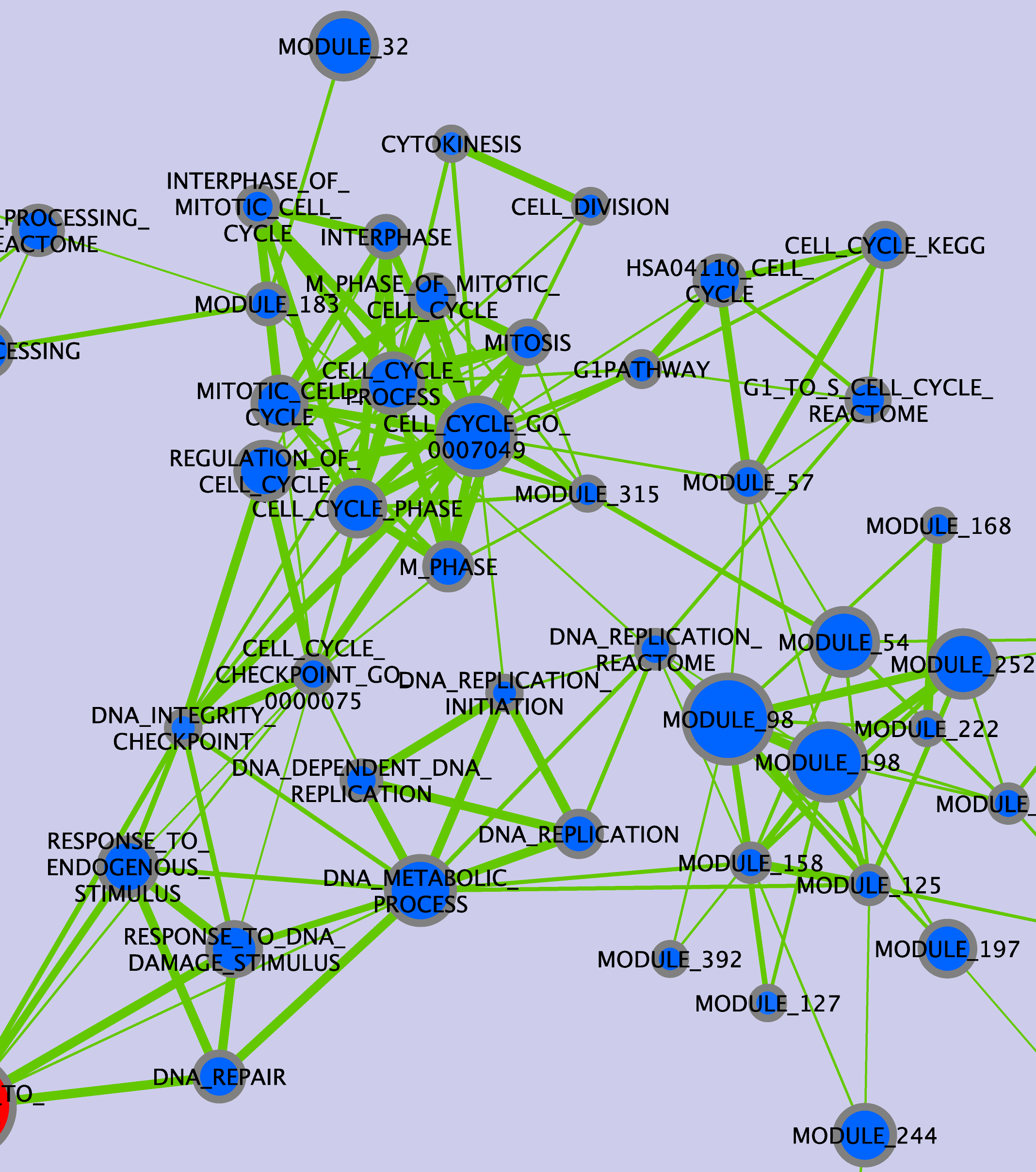


Figure 7. Sub-network from the enrichment map in Figure 3, including the *Cell cycle* and *DNA replication* clusters.


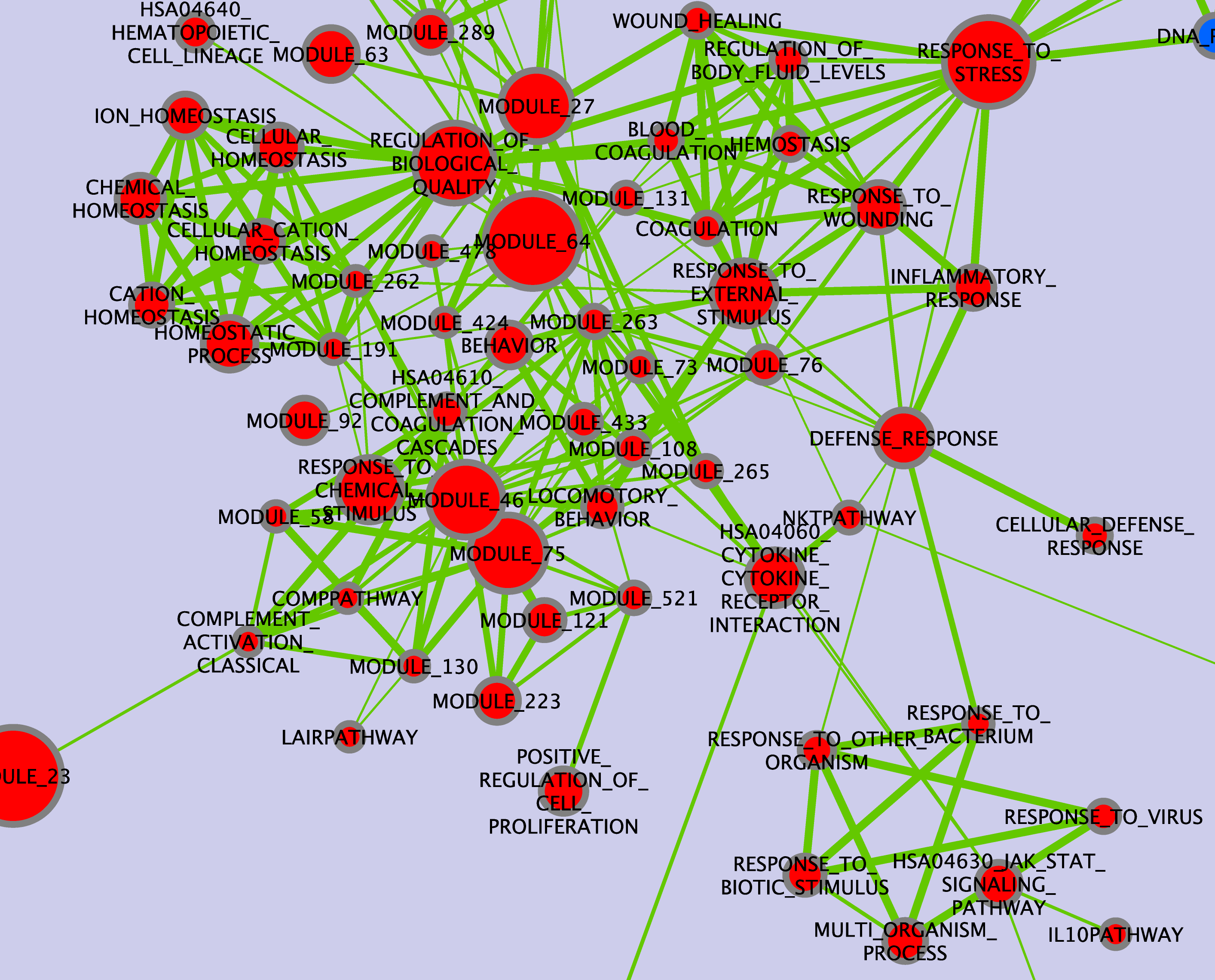


Figure 8. Sub-network from the enrichment map in Figure 3, including the *Ion homeostasis*, *Coagulation*, *Complement cascade* and *Response to pathogen* clusters.
